# Calculating earthquake damage building by building: the case of the city of Cologne, Germany

**DOI:** 10.1007/s10518-021-01303-w

**Published:** 2022-01-10

**Authors:** Cecilia I. Nievas, Marco Pilz, Karsten Prehn, Danijel Schorlemmer, Graeme Weatherill, Fabrice Cotton

**Affiliations:** 1 Seismic Hazard and Risk Dynamics, GFZ German Research Centre for Geosciences, Potsdam, Germany; 2 University of Potsdam, Institute for Geosciences, Karl-Liebknecht-Str. 24-25, 14476 Potsdam, Germany

**Keywords:** Building exposure modelling, Seismic damage assessment, Scenario earthquake, Seismic risk, Cologne

## Abstract

The creation of building exposure models for seismic risk assessment is frequently challenging due to the lack of availability of detailed information on building structures. Different strategies have been developed in recent years to overcome this, including the use of census data, remote sensing imagery and volunteered graphic information (VGI). This paper presents the development of a building-by-building exposure model based exclusively on openly available datasets, including both VGI and census statistics, which are defined at different levels of spatial resolution and for different moments in time. The initial model stemming purely from building-level data is enriched with statistics aggregated at the neighbourhood and city level by means of a Monte Carlo simulation that enables the generation of full realisations of damage estimates when using the exposure model in the context of an earthquake scenario calculation. Though applicable to any other region of interest where analogous datasets are available, the workflow and approach followed are explained by focusing on the case of the German city of Cologne, for which a scenario earthquake is defined and the potential damage is calculated. The resulting exposure model and damage estimates are presented, and it is shown that the latter are broadly consistent with damage data from the 1978 Albstadt earthquake, notwithstanding the differences in the scenario. Through this real-world application we demonstrate the potential of VGI and open data to be used for exposure modelling for natural risk assessment, when combined with suitable knowledge on building fragility and accounting for the inherent uncertainties.

## Introduction

The creation of building exposure models for seismic risk assessment is challenging, as relevant detailed information on structural properties, size and value of all the buildings in an area of interest is seldom readily available for such a use. Building-by-building surveys undertaken by qualified professionals are only possible within limited spatial extents, and wide-spread building censuses, though useful, are still relatively uncommon. They also tend to focus on a reduced number of variables, contain no or limited details on structural characteristics, and can only be carried out every certain number of years, given their resource intensiveness (e.g., Crowley et al. 2020; Yepes-Estrada et al. 2017). Exposure models are thus normally developed following a series of techniques that allow modellers to infer structural properties and characteristics of the building stock via proxies that might be more easily accessible. For example, the data from housing censuses can be combined with expert knowledge on the building classes that exist in a region to define a distribution of residential building classes from parameters such as the predominant materials of the walls and floors and the type of dwellings (e.g., Yepes-Estrada et al. 2017). Remote sensing techniques can be incorporated to the process in many ways, ranging from delineating areas of homogeneous urban structure or land use, either alone or combined with volunteered geographic information sources and/or street-level photos from omnidirectional cameras (e.g., Wieland et al. 2015), all the way through to the automatic recognition of building footprints (e.g., Geiß et al. 2017a). Automatic classification of buildings detected through remote sensing techniques into structural classes and the automatic assignment of missing attributes have been more challenging and tend to be resource-intensive in their need for expert human intervention for the collection of accurate in-situ information and/or labelling of datasets to be used for the training of the algorithms; several studies have yielded promising results in this regard (e.g., Geiß et al. 2015, 2017b; Liuzzi et al. 2019).

Volunteered geographic information (VGI) sources have become increasingly relevant for exposure modelling in recent years, whether combined with remote sensing techniques or as core datasets themselves. One of the most relevant examples of VGI in terms of activity, growth rate and coverage is OpenStreetMap (OSM), a community-based open data project in which contributors voluntarily map and describe geographic features, including buildings and roads. The features get characterised and described by means of so-called *tags*, which, in the case of buildings, provide information such as the kind of human activity they host (e.g., residential, commercial), the number of storeys, type of roof, and others. Though the spatial coverage of OSM is not uniform around the globe (e.g. Herfort et al. 2021), the number of buildings represented on the map keeps on increasing in time. OSM has been already successfully used in the development of not only residential exposure models but also industrial ones, which present their own very specific challenges (e.g., Geiß et al. 2017a; Sousa et al. 2017).

The methods, techniques and data sources employed are only one aspect of the development of exposure models for risk assessment. Another related aspect is associated with the spatial resolution of the resulting models. The generalised lack of detailed information about each building and the frequent use of census data to infer distributions of structural classes leads to models often being defined at an administrative unit level. Depending on the scale of the risk assessment and the size of the administrative units, risk analyses may subsequently be carried out directly by administrative unit or at a finer resolution achieved through the disaggregation of the models by means of ancillary data, such as open datasets of human settlements and/or population distributions (e.g., Dabbeek and Silva 2020), or even VGI (e.g., Figueiredo and Martina 2016). This aggregation implies that ground motions are calculated at one location intended to represent a much larger area, introducing potential inaccuracies in the characterisation of site effects as well as the distance (and relative position) between the seismic source and the assets, an issue that becomes particularly relevant in the near-field of large earthquakes where amplitudes of ground motion can change rapidly with distance from the fault. Risk analyses at the building-by-building level are still relatively uncommon, particularly when the study area is extensive, but are becoming of greater interest as computational capacities allow for more demanding calculations to be carried out and more data about buildings is being collected and becoming available. Building-by-building exposure models open the opportunity for a large number of scientific developments. Knowing the exact location of a building allows for an increased accuracy in the estimation of the ground motions at its base: such improvements can be relatively simple like a better estimate of the distance to the source, but also allow for the incorporation of high-resolution site amplification models, directionality and directivity effects, as well as full 3D wave-propagation models. The knowledge of precise asset locations and access to them can also help making planning and executing of emergency response more targeted and efficient. While focus here is placed on seismic action, high-resolution exposure models are relevant for all sorts of perils, particularly those in which the exact location of the buildings is a controlling factor (e.g. flood risk).

It is with this perspective in mind that this paper explores the development of a building-by-building exposure model through the combination of the advantages of OSM, VGI and open data in general. It integrates the most up-to-date information on the location of buildings and their use, as well as useful building-by-building parameters of relevance for characterising vulnerability such as year of construction and number of storeys, with other open data like statistics on year of construction aggregated at various administrative levels that can complement and improve the model. This combination of complete statistics collected throughout the previous decades with up-to-date but incomplete building-by-building data will be discussed in detail in the paper. We use Monte Carlo simulation to allocate specific properties (defined in a statistical sense) to specific buildings while accounting for the uncertainty associated with carrying out such an allocation, an approach that shares similarities with the work of Sieg et al. (2019) in the area of hydrometeorological risk.

Though applicable to any other city or region of interest where analogous datasets are available, the workflow and approach followed to develop a building-by-building exposure model are illustrated by focusing on the case of the German city of Cologne, going all the way through to the estimation of damage due to a scenario earthquake. Though Cologne is exposed to low-to-moderate seismic hazard within the context of Europe, it is located in an area with one of the highest levels of seismic hazard in Germany (Grünthal et al. 2018). Because of the large number and value of exposed assets, the highest losses due to seismic risk in the country with a 10% probability of exceedance in 50 years are expected in Cologne (Tyagunov et al. 2006). The earthquake scenario considered is that of a moment-magnitude *M*_*W*_ 6.5 rupture along the Erft fault, 18 km south-west of the geographical centre of the city. The potential losses due to this event are calculated for the residential building stock using the building-by-building exposure model developed herein.

The paper is split into two main parts: the first focuses on the development of the exposure model, while the second shows its use for the calculation of damage due to the scenario earthquake. As the creation of an exposure model goes hand in hand with the definition of the vulnerability of the assets exposed, the paper first discusses the modelling of the buildings’ fragility to seismic action as a means of identifying the parameters that are needed from the exposure model and that are available in the open datasets used. The merging of the datasets is described next, together with the main characteristics of the resulting initial model, from which we identify the need for the assignment of relevant parameters from other sources for a subset of the buildings. The following sections then describe the aggregated statistical distributions that are used to assign those parameters and the Monte Carlo approach adopted to do so, finally leading to a detailed description of the final building-by-building model and associated consistency checks. The second part of the paper describes the case study scenario, including its seismological aspects and the calculation of the ground motion and macroseismic intensity fields. We present results from the damage calculation and compare them against observed damage data resulting from the 1978 Albstadt earthquake, which is often named as one of the most damaging earthquakes recorded in Germany. Relevant findings, potentials and limitations of the procedure, the resulting model and the presentation of damage estimates are finally discussed.

## Characterising the assets at risk: building exposure and fragility

Any risk assessment results from the combination of three components: hazard (the external peril itself), exposure (the assets that exist in an area where the peril exists) and fragility/vulnerability (how susceptible the assets are to be affected by the peril). In the context of seismic risk, fragility describes the probability of a building resulting damaged to a certain degree in a scale when subject to a certain ground motion. The interface between hazard and fragility is a measure of ground motion intensity deemed relevant for the exposed assets (e.g. peak ground or spectral acceleration), while the interface between exposure and fragility is the classification of the assets (buildings) into groups with similar characteristics that result in similar likely consequences when affected by the hazard. This interface results in the existence of a two-way connection between exposure and fragility. The availability of fragility models in terms of certain building characteristics, such as a category within a classification schema or parameters such as the year of construction, dictates the need for an exposure model that identifies the existence and location of buildings in terms of those same characteristics (e.g. number of buildings built within a given time period in a particular neighbourhood). Conversely, however, the availability of certain building data (or lack of it) will drive the decision of which fragility model to use.

In this context, the decisions made in the present work for the creation of a building-by-building exposure model for the city of Cologne arose from a joint evaluation of the available datasets that could be used to characterise the exposure and the identification of a suitable fragility model that would best capitalise on information contained within these datasets. The fragility model is thus described first in the following sub-sections, to delineate the need for certain building attributes to be identified and used for the exposure model, whose development is subsequently explained.

### Building fragility

#### Vulnerability classes

The vulnerability classes of the European Macroseismic Scale (EMS-98) (Grünthal 1998), which range from A (most vulnerable) to F (least vulnerable), are a useful classification schema developed with a focus on European building typologies. According to the recommendations of Schwarz and Maiwald (2019), these classification schema can be linked in the city of Cologne to different time periods of construction, as shown in Table 1. These recommendations stem from the results of the German Research Network on Natural Disasters project (*Deutsches Forschungsnetz Naturkatastrophen* in German, DFNK hereafter), for which 631 buildings in the Cologne neighbourhoods of the Severins-Viertel and Georgs-Viertel were assessed in detail. Intermediate classes AB, BC and CD indicate intermediate vulnerability levels between the two indicated classes (“AB” means more vulnerable than B but less vulnerable than A, and so on).

**Table 1:** Distribution of EMS-98 vulnerability classes per time period of construction in the city of Cologne according to Schwarz and Maiwald (2019)

Time period of construction	EMS-98 vulnerability class						
	← More vulnerable Less vulnerable →						
	A (%)	AB (%)	B (%)	BC (%)	C (%)	CD (%)	D (%)
Before 1918	4.5	4.5	91.0	0	0	0	0
1919–1948	0	0	70.0	0	30.0	0	0
1949–1962	0	0	5.4	5.4	89.2	0	0
1963–1975	0	0	1.5	1.5	89.1	6.3	1.6
1976–1989	0	0	0	8.2	82.2	8.2	1.4
After 1990	0	0	0	0	88.9	11.1	0

#### Fragility model

The fragility model developed by Raschke (2003) describes the probability of observing a particular damage grade in the EMS-98 scale (Grünthal 1998) as a function of EMS-98 macroseismic intensity, *I*, and a vulnerability index, *C*. The vulnerability index depends mainly on the vulnerability class but can be refined according to other relevant parameters, such as the number of storeys, as recommended by Schwarz and Maiwald (2019) and done herein (Table 2). The EMS-98 damage scale goes from damage grade 1 (DG1), described as negligible-to-slight damage (no structural damage, slight non-structural damage), through to damage grade 5 (DG5), which corresponds to very heavy structural damage and even collapse. It is implicit that DG0 refers to no damage.

**Table 2:** Vulnerability index *C* to be used in the fragility model described herein

Number of storeys	EMS-98 vulnerability class						
	A	AB	B	BC	C	CD	D
Agnostic	*0.00*	*0.50*	*1.00*	*1.50*	*2.00*	*2.50*	*3.00*
1	0.49	0.99	1.49	1.99	2.75	3.25	3.75
2	*0.00*	*0.50*	*1.00*	*1.50*	2.41	2.91	3.41
3	− 0.46	0.04	0.54	1.04	*2.09*	*2.59*	*3.09*
4	− 0.85	− 0.35	0.15	0.65	*1.78*	*2.28*	*2.78*
5	− 1.18	− 0.68	− 0.18	0.32	1.50	2.00	2.50
6	− 1.49	− 0.99	− 0.49	0.01	1.29	1.79	2.29
7	− 1.49	− 0.99	− 0.49	0.01	1.00	1.50	2.00
8	− 1.49	− 0.99	− 0.49	0.01	0.80	1.30	1.80
9	− 1.49	− 0.99	− 0.49	0.01	0.64	1.14	1.64
≥ 10	− 1.49	− 0.99	− 0.49	0.01	0.52	1.02	1.52

Values shown for the case that is agnostic to the number of storeys are those of Raschke (2003), while those for specific numbers of storeys were recommended by Schwarz and Maiwald (2019), who derived them as part of the DFNK project from the earthquake damage data of Raschke (2003). Values marked in italics show the number of storeys or range of number of storeys that are represented by the agnostic case for each vulnerability class (e.g., for class C, the agnostic case indicates C = 2.00, which lies between 1.78 and 2.09, i.e. equivalent 3-to-4 storeys)

**Fig. 1:**
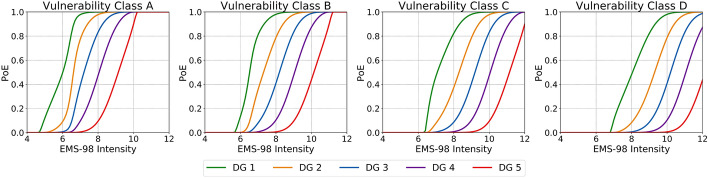
Probabilities of exceedance (PoE) of each EMS-98 damage grade (colour scale) resulting from the model of Raschke (2003), using vulnerability indices agnostic to the number of storeys

The model comprises two main steps, the first one related to determining the mean damage grade *D*_m_ and a second one in which *D*_m_ is converted into a set of probabilities of observing each of the possible damage grades from DG0 through DG5. Details on these steps and the background of the model can be found in Appendix A. Figure 1 depicts the resulting probabilities of exceedance of each EMS-98 damage grade (fragility curves) for four different vulnerability classes, obtained using the values shown in Table 2 for an unspecified number of storeys. It is noted, though, that including the number of storeys can result in significant variations of these curves.

### Building exposure

#### Overview

The fragility model just described requires the exposure model to contain information on the time period of construction and number of storeys of the buildings that make up the study area—the city of Cologne in our application case. Additionally, the exposure model needs to enable the identification of the occupancy types of the buildings, as the fragility model of Raschke (2003) can only be assumed to represent standard structural types and not special kinds such as churches, hospitals or schools, whose structural characteristics can be very diverse and their seismic response very difficult to define without a more detailed investigation. The empirical data used by Raschke (2003) seem to refer to entire building stocks, of which special buildings can only be expected to represent a small fraction. Similarly, the test area within the city of Cologne from which the link between time period of construction and vulnerability classes was derived (Table 1) appears to be rich in residential and mixed residential-commercial structures, but not as much in other kinds. For these reasons, the focus in the present study was set upon residential and mixed residential-commercial buildings, the whole set of which is named as “residential” for brevity in what follows.

**Fig. 2:**
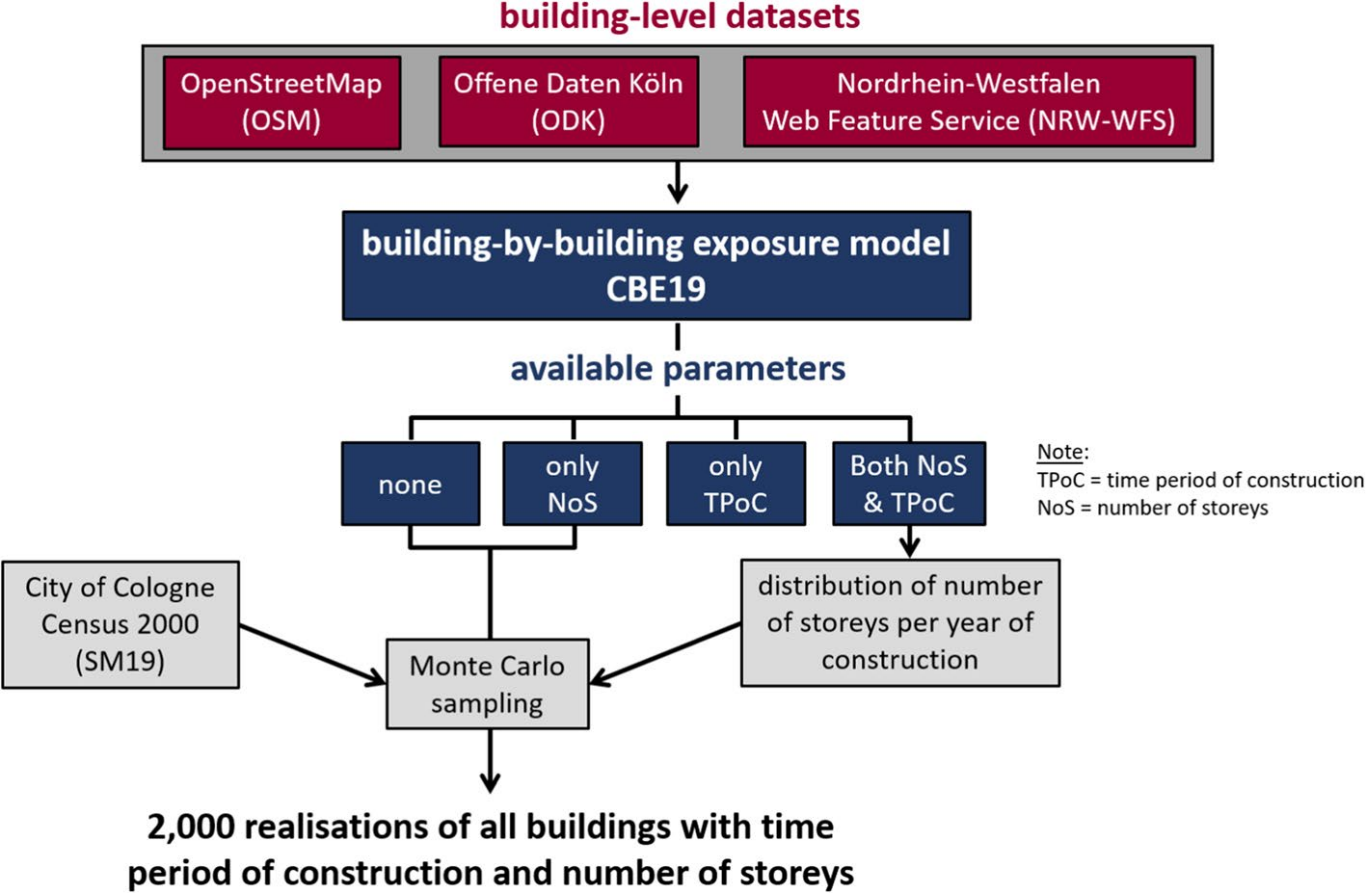
Simplified schematic representation of the process followed to generate the building exposure model for the city of Cologne. SM19: Schwarz and Maiwald (2019)

We developed a building exposure model for the city of Cologne using a two-fold strategy that aimed at taking maximum advantage of data available at the building-by-building level and complementing it with independent statistics, which are aggregated at the neighbourhood (*Viertel*, in German) scale. As shown schematically in Fig. 2, the three openly-available sources that made it possible to work at the building-by-building level were:

1. OpenStreetMap (OSM), an open database of geographic information/features collected by volunteers,
2. a dataset containing information on years of construction, available from *Offene Daten Köln* (Stadt Köln 2019; ODK hereafter), an open-contribution web service from the city of Cologne, and
3. a dataset containing information on the number of storeys and occupancy, available from the Nordrhein-Westfalen Web Feature Service (2019), that provides cadastral information for the whole state (NRW-WFS hereafter).

The merging of these three datasets and the classification of buildings into occupancy cases was the first step in the process of building the exposure model. As not all residential buildings could be assigned a time period of construction and/or number of storeys by the end of this first step, this (building-by-building) Cologne Building Exposure (CBE19) model was enriched in a second step by data aggregated at the city and neighbourhood levels. This data stems from two main sources: (1) the distribution of time periods of construction per neighbourhood surveyed by the city of Cologne in the year 2000 and summarised by Schwarz and Maiwald (2019), and (2) the distribution of number of storeys per time period of construction derived from the building stock for which both parameters were available in CBE19. Pre-processing of the statistics from the year 2000 was needed to account for the passage of 19 years as well as inconsistencies between the statistics and the building-by-building model resulting from the first step. As shown in Fig. 2, the assignment of numbers of storeys and/or time periods of construction to buildings without such information was carried out by means of a Monte Carlo simulation. All details on these and all the sub-steps followed to generate the exposure model are given in this section.

#### Initial building-by-building exposure: merging datasets

**Fig. 3:**
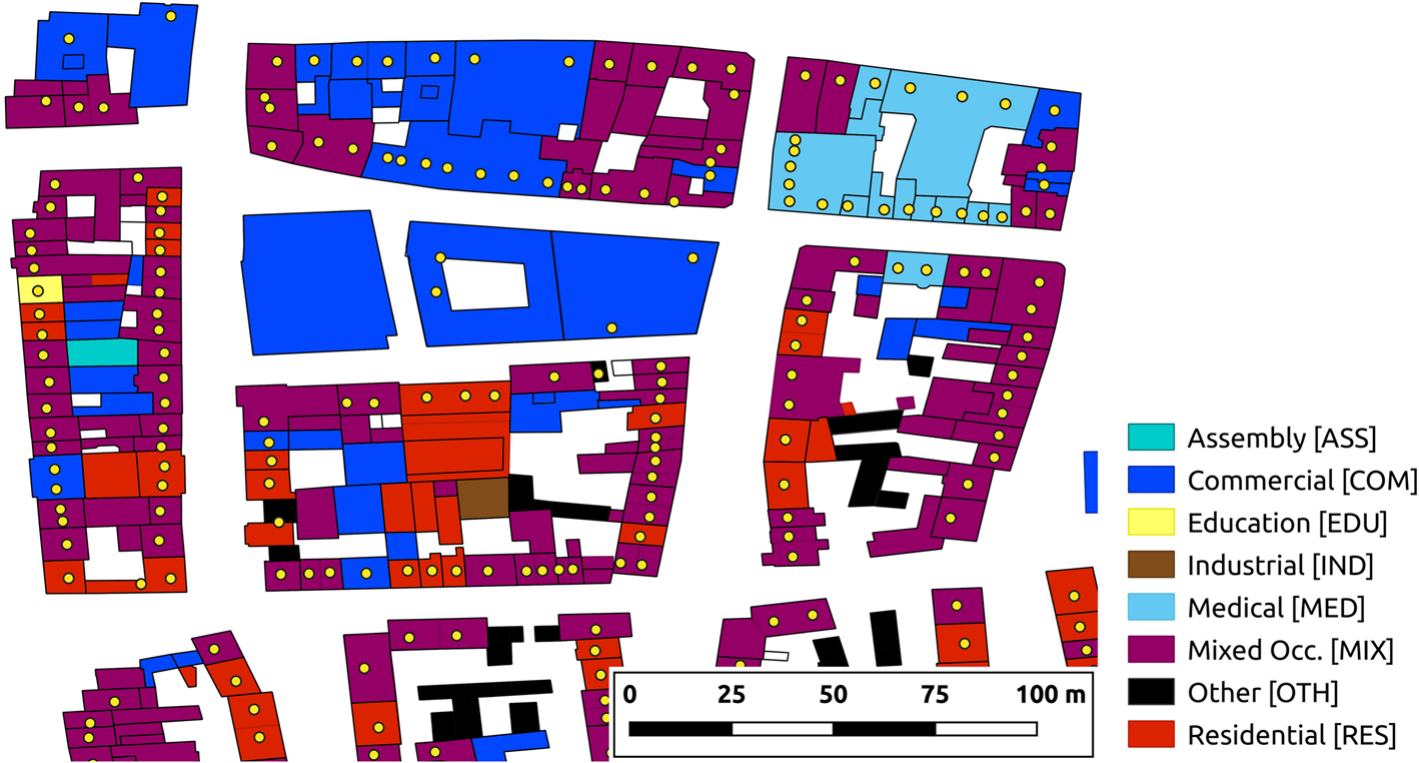
Example of overlap of the three datasets used to generate the building-by-building exposure model for Cologne: OpenStreetMap (filled polygons), NRW-WFS (black polygon contours), and ODK (yellow dots). The different polygon colours indicate the occupancy category finally assigned

The first step to generate the building-by-building exposure model was to merge the information from the three datasets mentioned above, which originally covered buildings of all occupancy kinds and not just residential ones. In order to do so, OSM polygons tagged as buildings, polygons from NRW-WFS and longitude-latitude points from ODK needed to be matched with one another to identify all pieces of information associated with each building. Both the ODK and the NRW-WFS datasets were compared against OSM first by means of geospatial intersection and then through the addresses retrieved from both, ODK and NRW-WFS. The latter made it possible, for example, to match a point from ODK that fell slightly outside of an OSM polygon through the address of the NRW-WFS polygon that had been matched with the OSM target, and vice versa. Figure 3 shows an example of the overlap of the three datasets. These and other decisions associated with the combination of the three input datasets were informed by imagery from Google Maps, Google Earth, Google Street View and Mapillary. Further details on the strategy followed to carry out the merging can be found in Pilz et al. (2020b).

The assignment of occupancy types was relevant to be able to classify buildings into residential and non-residential. This was done using several sources in a hierarchical scheme, with the highest priority given to NRW-WFS strings associated with the building polygons that describe the function of the building (in German; a mapping dictionary to assign occupancy types from these was created and is presented as Appendix I of Pilz et al. 2020b), followed by OSM points of interest (i.e., nodes indicating the existence of, for example, a bakery, a school, a hospital, etc.), followed by OSM building tags (e.g. “amenity”, “tourism”, “leisure”) and then OSM land-use layers (i.e., polygons encircling areas of uniform land use), with areas of land use from the CORINE Land Cover project (European Environmental Agency, EEA) employed as a last resort. All these data were processed to assign occupancy classes based on v2.0 of the Building Taxonomy of the Global Earthquake Model (GEM; Brzev et al. 2013), which was expanded to separate categories that were deemed to deserve their own classes.

While the level of coverage of OSM is variable around the world, the building footprint data in the administrative unit of the city of Cologne can be considered complete, with 286,373 buildings identified by 21st August 2019. Within the same administrative unit, the ODK dataset comprises 159,994 points with year of construction, while the NRW-WFS dataset consists of 301,120 polygons classified as “Gebäude” (building) or “Bauwerk oder Anlage für Industrie und Gewerbe” (building or plant for industry and commerce). Of the 286,373 buildings identified in OSM, 147,388 (51.5%) have been classified as residential, while 22,083 (7.7%) have been identified as MIX1 (mixed residential and commercial). Further development of the exposure model and damage calculations have focused on these 169,471 buildings. The complete classification of all buildings according to their occupancy is presented in Appendix B. Information on both number of storeys and year of construction is known for 129,349 (76.3%) of these 169,471 residential buildings that make up the CBE19 model, while a great majority of the remaining buildings is associated only with data on the number of storeys, as shown in Table 3.

**Table 3:** Number and percentage of residential buildings in the CBE19 model classified as per the availability of time period of construction and number of storeys

Subset	Number	%
Both time period of construction and number of storeys	129,349	76.3
Only time period of construction	939	0.6
Only number of storeys	34,439	20.3
Neither time period of construction nor number of storeys	4744	2.8

#### Enrichment with aggregated statistics

As around one quarter of the residential buildings are missing a time period of construction and/or number of storeys, the next steps aim at assigning these parameters to such buildings by means of Monte Carlo simulations, as schematically shown in Fig. 2. In broad terms, each of the following sub-sections deals with (1) adjusting statistics of time periods of construction available for each neighbourhood for the year 2000, based on incomplete statistics for the year 2019, (2) defining distributions of numbers of storeys per time period of construction for the whole city, and (3) combining the output of (1) and (2) to generate theoretical joint distributions of time period of construction and number of storeys for each neighbourhood, adjusting them to accommodate discrepancies with values observed in CBE19, and using these adjusted distributions to assign time period of construction and/or number of storeys to those buildings for which these parameters were not available.

##### Definition of theoretical distributions of time periods of construction

The most relevant source of aggregated data used to enrich the building-by-building CBE19 model was information on the distribution of time period of construction per neighbourhood surveyed by the city of Cologne in the year 2000 and summarised by Schwarz and Maiwald (2019). Given the almost two-decade gap in between these statistics and CBE19, this distribution needed to be adjusted to reflect the corresponding increase in the number of buildings and the expected larger proportion of post-2000 buildings. In order to do this, a first check was carried out at the city level, by comparing total numbers of buildings with residential space stemming from two different censuses from the years 2000 and 2011 against the 169,471 buildings identified in the CBE19 model (Table 4). This first check revealed an average annual increase in the overall number of buildings with residential space of around 0.6% when comparing the total 128,971 contained in the dataset of Schwarz and Maiwald (2019) for the year 2000 against the 137,725 stemming from the 2011 German National Population and Housing Census (German Federal Statistical Office). The same average annual increase could be calculated from data by the city of Cologne for the period 2000–2015 (Stadt Köln 2016). However, the 169,471 buildings with residential space identified in the CBE19 model would imply an average annual increase of 2.6% since 2011. This significant change in annual increase is unlikely to be completely real and may reflect limitations of the input datasets and the algorithms implemented to process them, as well as potential discrepancies between the datasets and the census definition of what are considered buildings “with residential space” (particularly in the case of mixed use). Cases have been observed of buildings that look like one entity on street-level photos but are represented as more than one polygon in OSM and/or NRW-WFS, which may lead to the existence of more than one polygon per census building. At the same time, misalignments between the three different datasets have been solved algorithmically (a case-by-case human-based decision is not possible when dealing with large datasets), which means that the potential exists, for example, for a building to have been assigned a mixed residential-commercial occupancy (hence counted in this model) when it is in fact purely commercial (not counted in the census). As no further considerations can be made from the available information, the 169,471 residential buildings were kept in the CBE19 model, acknowledging the number might represent a slight overestimation.

**Table 4:** Number of buildings with residential space (including purely residential and mixed residential-commercial use) in the city of Cologne

Year	Source		Number of buildings		
			Before 1990	After 1990	Total
2000	Schwarz and Maiwald (2019)		116,279	12,692	128,971
2011	2011 German Census		114,607	23,118	137,725
2019	CBE19	Known year of construction	103,579	26,709	–
		All	*116,282*^(*)^	*53,189*^(*)^	169,471

Numbers marked with (*) were not known a priori and have been calculated by the procedure described in the main text

**Fig. 4:**
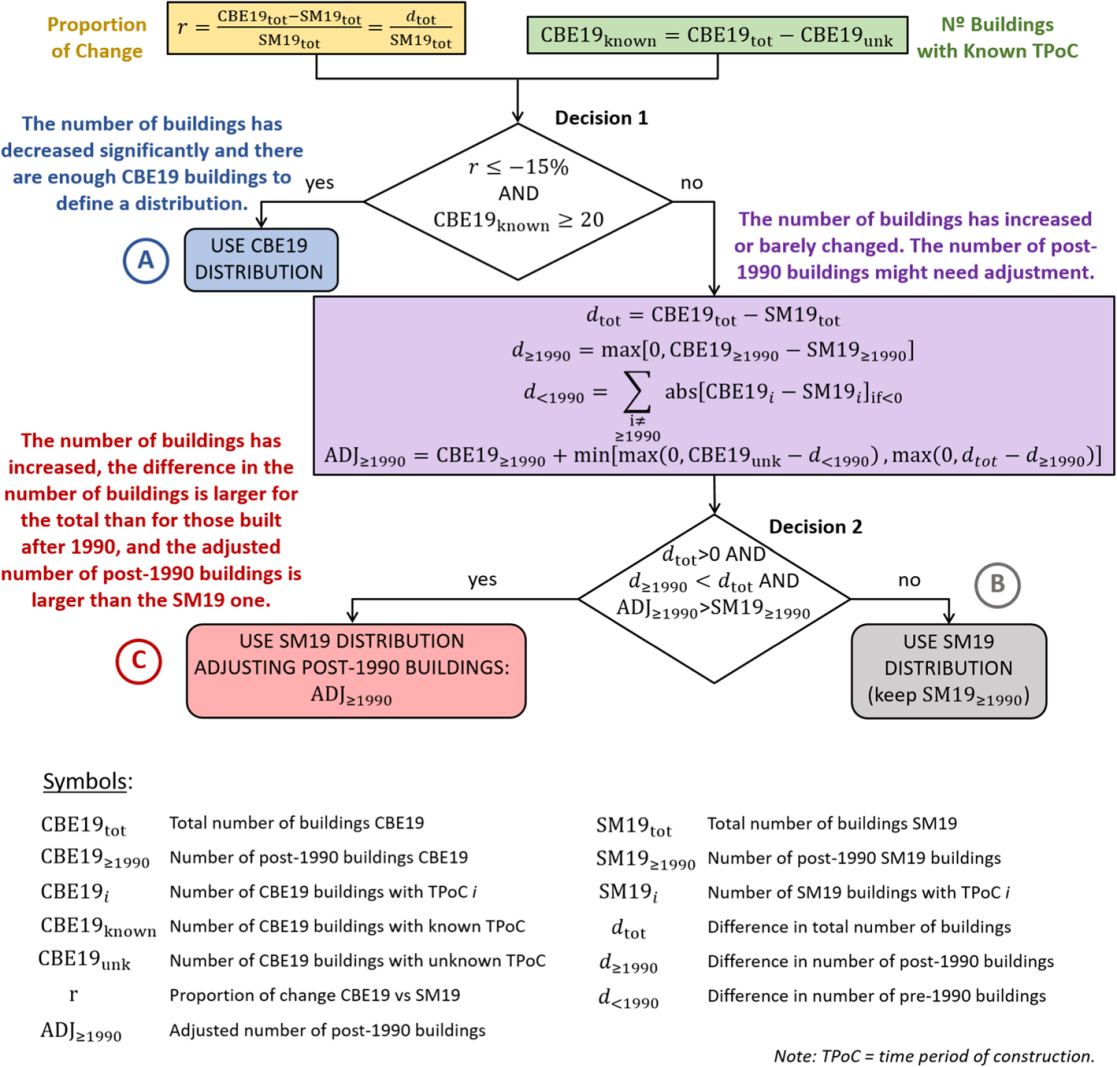
Procedure followed to define the theoretical distributions of time period of construction as a function of the statistical data for the year 2000 (Schwarz and Maiwald 2019; SM19) and the time period of construction of the present model (CBE19) for each neighbourhood

The adjustment of the distributions of time periods of construction per neighbourhood was challenging due to the fact that the years of construction are not available for all buildings and it is not possible to know a priori how the number of buildings may have evolved in terms of demolitions and new constructions. When comparing the data by neighbourhood, we observed that the distributions for the year 2000 (City of Cologne; Schwarz and Maiwald 2019) and those of the known buildings of the CBE19 model could be quite different, and that in many neighbourhoods the total number of buildings in 2019 appears to be much smaller than that reported for the year 2000. These observations and a detailed analysis of a few selected neighbourhoods as described in Pilz et al. (2020b) shaped the procedure followed to define the *theoretical distributions of time periods of construction* per neighbourhood, whose conceptual flowchart is depicted in Fig. 4.

The first decision was whether to adopt the *time period of construction (TPoC)* distribution stemming from the present model (CBE19) or consider the distribution for the year 2000 (SM19 in Fig. 4). The distribution stemming from CBE19 was adopted when the following two criteria were simultaneously satisfied:

1. the ratio of change in the total number of buildings from 2000 to 2019 (*r*) was less than −15%, which indicates a significant decrease in the number of buildings in time, and
2. the number of CBE19 buildings for which the time period of construction is known (CBE19_known_) was greater than 20.

The core assumptions behind these criteria are that if the number of CBE19 buildings is much smaller than that of SM19 buildings, then the SM19 distribution may not really be applicable because if the change is due, for example, to demolitions, it is not possible to know if the distribution of the year 2000 was preserved. The thresholds of a *r* ≤ −15% change (i.e., the number of CBE19 buildings is smaller than that of SM19 buildings by at least 15%) and CBE19_known_ ≥ 20 buildings were selected by visual inspection of the data, as well as considering that there are six categories of time period of construction (i.e., 20 buildings allows for an average 3.3 buildings per range). Out of 360 neighbourhoods, eight fell under this case (marked in blue and with the letter A in Fig. 4) and the CBE19 distribution was used for them as the theoretical distribution.

For those neighbourhoods for which the criteria defined above were not satisfied (either because *r* was larger than −15% or CBE19_known_ was smaller than 20), the SM19 distribution was used, and it was decided as per the steps that follow whether an adjustment for the passage of time between the years 2000 and 2019 was appropriate or not. If the data were perfect (i.e., if there were no inconsistencies across sources), no demolitions occurred and the time period of construction were known for all CBE19 buildings, the difference between the total number of CBE19 and SM19 buildings should then be equal to the number of buildings built after the year 2000 and, thus, after 1990, which is the last category considered for the assignment of vulnerability classes (Table 1). However, such an inference was difficult to make when having a proportion of CBE19 buildings with unknown years of construction and reductions in the number of buildings built before 1990, as it cannot be established if the buildings with unknown time period of construction corresponded to pre-1990 or post-2000 buildings.

As shown in Fig. 4, the criterion used for deciding the need for adjustment (“Decision 2”) was based on checking, firstly, if the number of CBE19 buildings was larger than that of SM19 buildings (i.e., *d*_tot_ > 0) and, secondly, if the difference in the number of buildings between CBE19 and SM19 was larger for the total than for those built after 1990 (*d*_≥1990_ < *d*_tot_). If these conditions were met, then the number of buildings built after 1990 was adjusted (ADJ_≥1990_); otherwise, the number of buildings built after 1990 was taken as that from SM19 (SM19_≥1990_). The adjustment (i.e., the calculation of ADJ_≥1990_ in Fig. 4) was carried out accounting not only for those CBE19 buildings known to have been built after that date but also for the largest number of buildings with unknown time period of construction that could be allocated to the post-1990 category without contradicting the CBE19 observations. In other words, we accounted for the fact that some of the additional buildings *d*_tot_ that may have been built after 1990 could potentially be already counted in *d*_≥1990_ while some others might remain unknown. Consequently, it cannot be assumed that the number of post-1990 buildings can be increased with respect to the CBE19 value more than by the number of buildings with unknown time period of construction. Moreover, some of the CBE19 buildings for which the time period of construction is not known may correspond as well to time periods of construction other than post-1990, if the number of buildings in these other ranges was smaller in CBE19 than in SM19. The summation of the absolute value of the differences between the CBE19 and the SM19 number of buildings for all ranges other than post-1990 across the cases in which the difference is negative, designated as *d*_<1990_ in Fig. 4, accounted for this. Finally, the calculated adjusted value should not be smaller than the number of buildings built after 1990 according to SM19. In the algorithm and, consequently, Fig. 4, this last condition (i.e., ADJ_≥1990_ > SM19_≥1990_) was verified together with the previous two (see the rhombus labelled as “Decision 2”). Out of the 352 neighbourhoods for which the CBE19 distribution was not adopted, 97 were assigned the SM19 distribution (marked in grey and with the letter B in Fig. 4) and 255 the corresponding adjusted version (marked in red and with the letter C in Fig. 4).

The output of the algorithm just described, which was run for each of the 360 individual neighbourhoods of Cologne, was the adopted *theoretical distribution of time periods of construction* for each neighbourhood, be it that resulting from the ends labelled as A, B or C in Fig. 4. Situations in which the number of buildings built before 1990 increased in CBE19 with respect to SM19 were not solved at this stage of defining the theoretical distributions but rather during the process of assigning time periods of construction and/or numbers of storeys to buildings as will be explained shortly. Such cases are a problem of inconsistency between datasets and may be potentially related to whether a polygon in the datasets represents one or more buildings, or whether one building is represented by one or more polygons, and how buildings were counted by the city of Cologne when carrying out the census in the year 2000 (for example, the question of whether one building is equivalent to one address or one structure).

##### Distribution of number of storeys per time period of construction

In order to be able to use the theoretical distributions of time periods of construction per neighbourhood to assign time periods of construction to buildings for which this information was not available, it was necessary to impose certain constraints to avoid non-realistic assignments. For example, it is highly unlikely that a 30-storey building in Cologne was built before 1918. This issue was addressed by retrieving the relationship between time period of construction and number of storeys from the buildings for which both these parameters were already available (129,349 of the total 169,471 residential buildings) (Table 5), and combining this information with the theoretical distributions of time periods of construction (defined in the previous section) to produce a theoretical joint distribution of time periods of construction and number of storeys for each neighbourhood.

**Table 5:** Number of buildings per time period of construction and number of storeys for the 129,349 residential buildings for which both parameters are available in the CBE19 model

Time period of construction	Number of storeys									
	1	2	3	4	5	6	7	8	9	≥ 10
Before 1918	1388	3583	3020	2596	798	84	*(11) 4*	*(1) 0*	0	*(1) 0*
1919–1948	3591	10,166	2452	1897	354	55	8	*(1) 0*	0	0
1949–1962	5729	13,757	5230	4609	1455	376	125	45	43	15
1963–1975	4945	12,800	3320	2653	1058	553	224	*(274) 275*	131	179
1976–1989	4197	8275	1335	981	432	182	69	22	11	41
After 1990	5624	13,655	3558	2092	979	259	81	19	2	8

Cells marked in italics have been adjusted as described in the text (old unused number shown inside parentheses)

The existence of a few relatively tall old buildings in Table 5 prompted a manual verification of potential outliers. Starting with the case that appeared as the most unlikely on the basis of engineering judgement and knowledge of the area under study (10+ storeys built before 1918), the 14 buildings classified as being particularly tall and old (shown in italics in Table 5) were identified and their façades were analysed by means of street-level images from Google Street View and Mapillary, as well as the aerial 3D view of Google Maps. For ten of these 14 cases it was concluded that the time period of construction and/or number of storeys were incorrect in the original datasets. In nine cases the buildings were eliminated from the table, as shown in the cells marked in italics in Table 5, because more appropriate parameters could not be determined from the available information, while the one 8-storey building assigned as built between 1919 and 1948 was re-classified as being built in 1970. This was possible thanks to the aerial 3D view of Google Maps revealing not only that the building was more modern than specified in the ODK dataset (which indicated that it had been built in 1929), but also that it looked identical to another building within the same plot of land, for which ODK indicates 1970 as the year of construction. Two out of the eight 7-storey buildings assigned to the period 1919–1948 were investigated as well. In both cases the number of storeys and year of construction appeared as reasonable, indicating that this is not an unfeasible combination. The search was consequently stopped and no further combinations were scrutinised. While the accuracy of more common combinations of number of storeys and time period of construction was not assessed, it is expected that with more abundant numbers of buildings the number of misclassifications be small in proportion to the number of buildings in that combination and that the resulting error be tolerable and not systematic. Further details on this assessment process can be found in Pilz et al. (2020b).

The adjustments carried out are marked in italics in Table 5, while the resulting proportions of numbers of storeys per range of year of construction are presented in Table 6. As can be observed, each row adds up to 100%, as the purpose of this table is not to aid in assigning time periods of construction but to indicate the distribution of number of storeys associated with each time period group.

**Table 6:** Adopted distribution (%) of number of storeys per time period of construction

Time period of construction	Number of storeys									
	1	2	3	4	5	6	7	8	9	≥ 10
Before 1918	12.10	31.23	26.32	22.63	6.96	0.73	0.03	0.00	0.00	0.00
1919–1948	19.39	54.88	13.24	10.24	1.91	0.30	0.04	0.00	0.00	0.00
1949–1962	18.25	43.83	16.66	14.69	4.64	1.20	0.40	0.14	0.14	0.05
1963–1975	18.92	48.97	12.70	10.15	4.05	2.12	0.86	1.05	0.50	0.68
1976–1989	27.00	53.24	8.59	6.31	2.78	1.17	0.44	0.14	0.07	0.26
After 1990	21.40	51.96	13.54	7.96	3.73	0.99	0.31	0.07	0.01	0.03

##### Algorithm for the assignment of time period of construction and number of storeys

**Fig. 5:**
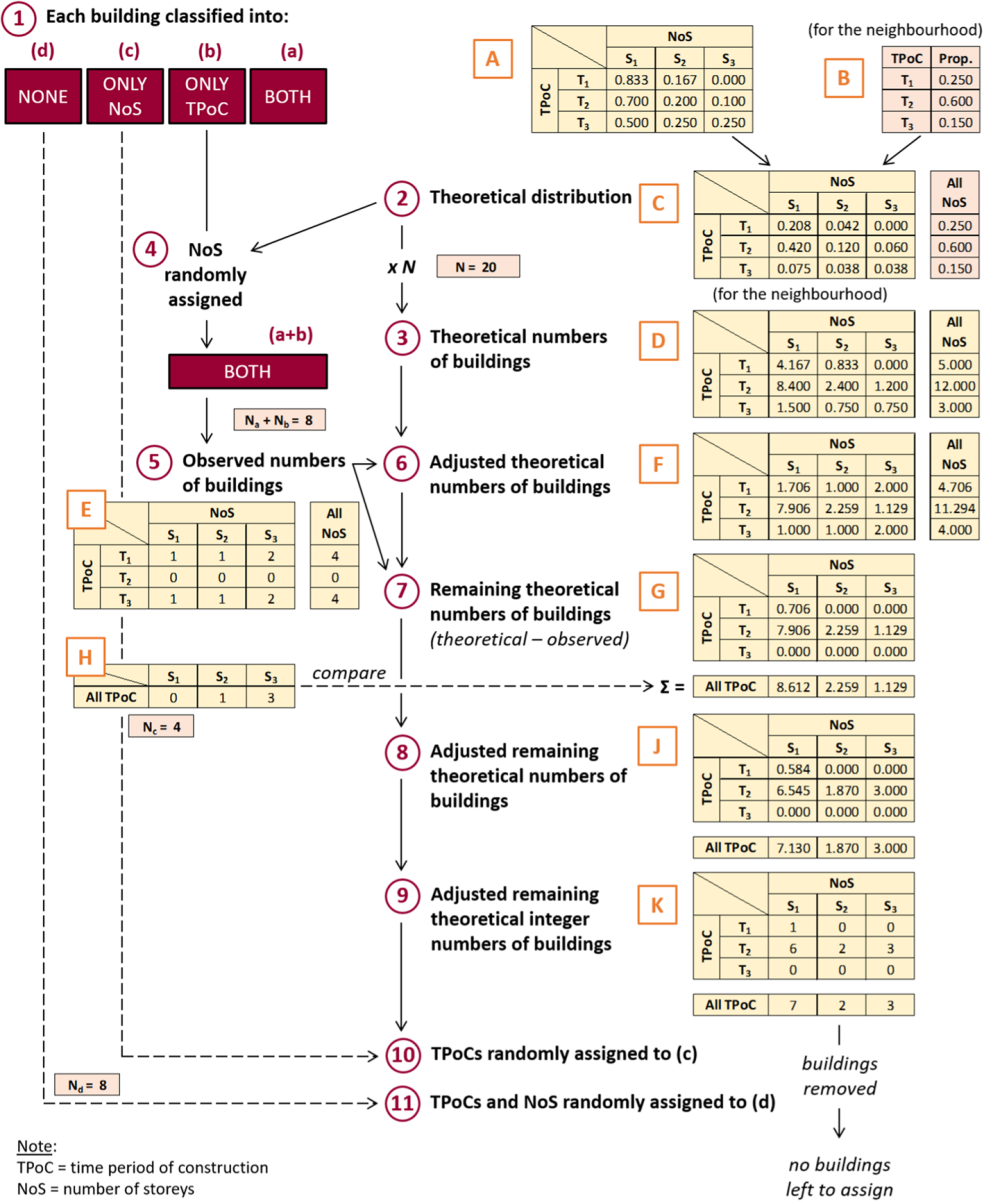
Schematic representation and simplified worked example of the steps followed to assign time periods of construction and/or numbers of storeys to the buildings for which these data were not available, for each neighbourhood. Red numbers correspond to steps enumerated in the main text

Making use of the theoretical distributions of time periods of construction and the distribution of number of storeys per time period of construction defined above, we assigned time periods of construction and/or numbers of storeys to the buildings for which these data were not available. For each neighbourhood, the procedure was as depicted in Fig. 5, which illustrates the steps via a simplified numerical example that makes use of three generic time periods of construction (T_1_, T_2_, T_3_) and three generic categories of numbers of storeys (S_1_, S_2_, S_3_). The numbers in Fig. 5 correspond to each of the following steps:

1. *Grouping* All buildings are grouped into four subsets:
  - buildings with both time period of construction and number of storeys;
  - buildings with only time period of construction;
  - buildings with only number of storeys;
  - buildings with none of the two parameters.

2. *Theoretical distribution* A matrix with the distribution of buildings by time period of construction and number of storeys for the neighbourhood is created using the aggregated distribution of years of construction (the outcome of Fig. 4, matrix B in Fig. 5) and Table 6 (matrix A). In practical terms this means that each row of matrix A is multiplied by the corresponding proportion of buildings falling into that time period of construction, to obtain matrix C.
3. *Theoretical numbers of buildings* Matrix C is multiplied by the total number of buildings in the neighbourhood (N), resulting in a matrix with the theoretical number of buildings per time period of construction and number of storeys (matrix D).
4. *Assigning number of storeys to subset (b)* The *theoretical distribution* matrix C obtained in step (2) is used to randomly assign numbers of storeys to buildings in subset (b), using the distribution of number of storeys corresponding to the known time period of construction. Subset (b) is treated in the same way as subset (a) from this point onward.
5. *Observed numbers of buildings in subsets (a) and (b)*: Subsets (a) and (b) are classified according to their time period of construction and number of storeys. This results in matrix E, which is analogous to matrix D but concerning observations instead of theoretical expected numbers.
6. *Adjusted theoretical numbers of buildings* The *theoretical numbers of buildings* from matrix D are compared against the *observed numbers* from matrix E. Whenever the number of buildings in a particular combination of time period of construction-number of storeys is larger in matrix E than in D, the theoretical numbers in D are adjusted by fixing the larger counts to the observations and re-distributing the remaining number of buildings proportionally to the theoretical distribution. This process is exemplified in detail in Fig. 6, where the cells whose values are fixed to those of matrix E are highlighted in orange. The purpose of this adjustment is to be able to maintain the theoretical distribution of time period of construction in the neighbourhood while at the same time allowing to account for what is observed in subsets (a) and (b). The outcome of this step is an adjusted theoretical number of buildings per category of time period of construction and number of storeys (matrix F).

**Fig. 6:**
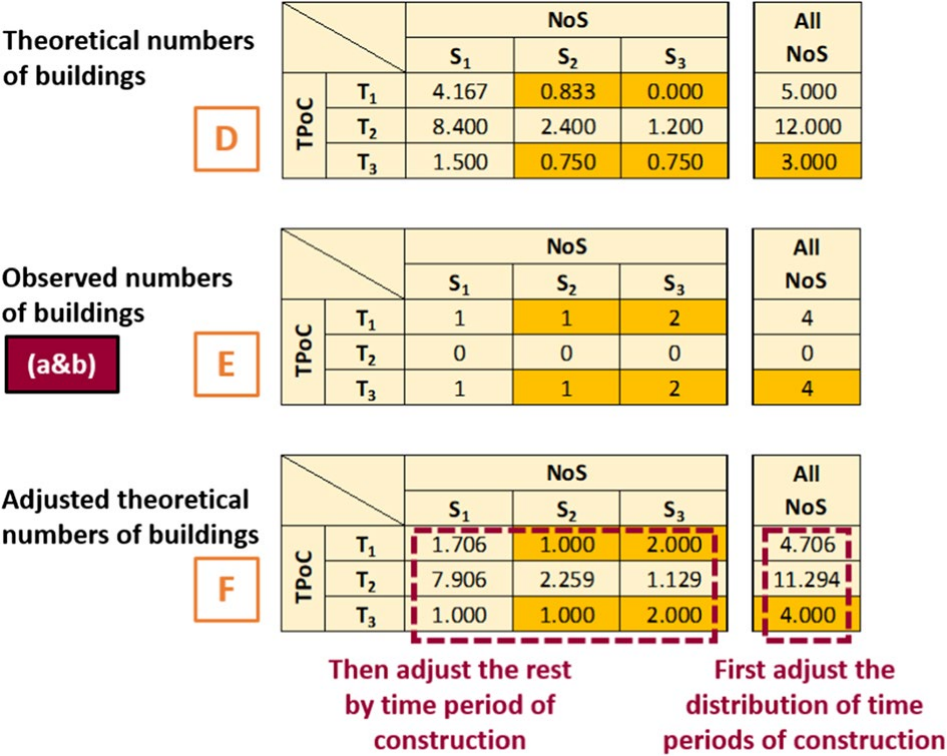
Simplified worked example of step 6. Whenever the observations in matrix E are larger than the theoretical numbers of buildings in matrix D, the numbers from matrix E are adopted (cells highlighted in orange). The remaining cells are adjusted proportionally. The overall distribution of time periods of construction (i.e., the summation per row) is adjusted first, followed by adjusting the individual cells in the matrix. The result is matrix F
7. *Remaining theoretical numbers of buildings* The buildings from subsets (a) and (b), presented in matrix E, are subtracted from the adjusted matrix F. This new matrix G represents only the buildings belonging to subsets (c) and (d).

8. *Adjusted remaining theoretical numbers of buildings* The new matrix G of *remaining theoretical numbers of buildings* resulting from step (7) is compared, grouped by number of storeys (summation per column), against subset (c) (matrix H), as shown in more detail in Fig. 7. If the number of observations from matrix H exceeds the theoretical number from matrix G, the matrix is adjusted by fixing the larger counts to the observations (in terms of total number of buildings per category of number of storeys) and re-distributing the remaining buildings across the other categories. If the number of observations in matrix H is non-zero for a certain category of number of storeys but the summation for that category is zero in matrix G, the distribution of time periods of construction in matrix G considering all number of storeys together is used for that category, forcing to zero any case of number of storeys incompatible with Table 6 (example, 8 storeys for a pre-1918 building). The outcome of this step is matrix J, which still contains theoretical numbers of buildings but now ensured to be compatible with the number of buildings per category of number of storeys from subset (c).

**Fig. 7:**
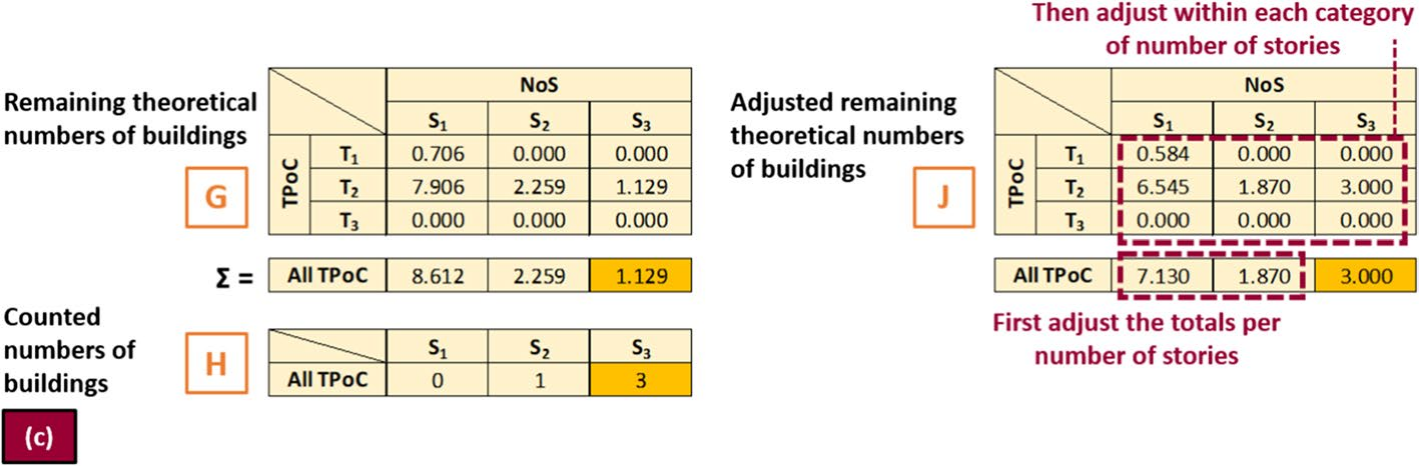
Simplified worked example of step 8. The observed numbers of buildings in subset (c) are compared against the summation per columns of matrix G (i.e. numbers of buildings per category of number of storeys). Numbers that are larger in H than in G are adopted for J. The remaining totals per column are adjusted proportionally first, before adjusting the individual cell contents to finally yield matrix J
9. *Adjusted remaining theoretical integer numbers of buildings* Matrix J of *adjusted remaining theoretical numbers of buildings* resulting from (8) is rounded from real numbers into integers. As this can lead to an artificial drop or increase in the total number of buildings, the necessary adjustments are made by adding/subtracting buildings to cells with the greatest discrepancy between the integer and the real numbers, as shown in the simplified example in Fig. 8. This is done first by category of number of storeys, comparing against matrix H, and then for the whole matrix. The output is matrix K.

**Fig. 8:**
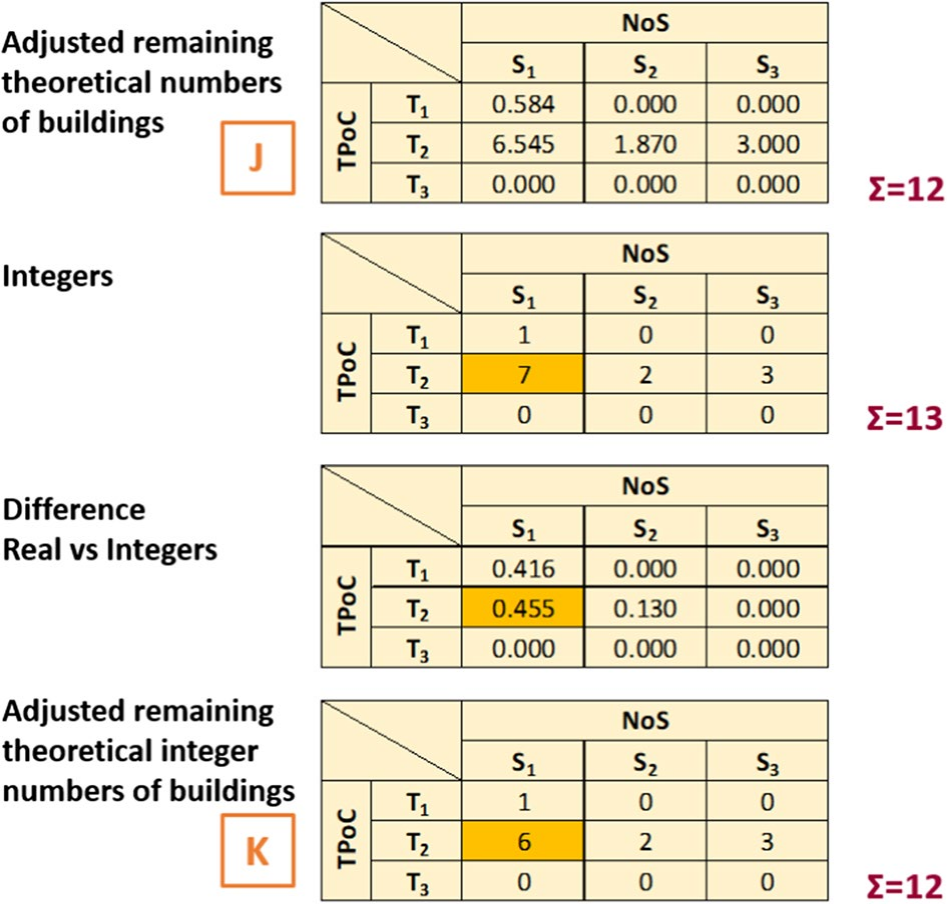
Simplified worked example of step 9. A simple rounding of the numbers in J to make them integers causes the total number of buildings to change (from 12 to 13). A building is then added or subtracted (depending on whether the total number of buildings has decreased or increased) to the cells with the largest discrepancy between the real numbers and their rounded integers, as shown in the highlighted cells. The process is repeated until the total number of buildings in K is the same as in J (the check is also carried out in the summation by column, as the summation per column of K still needs to yield numbers equal to or larger than those in matrix H; this detail is not shown here for simplicity)

10. *Assignment of time periods of construction to buildings in subset (c)* Each of the buildings in subset (c) is randomly assigned a time period of construction from matrix K. Each time a building is “assigned”, it is eliminated from the matrix, which always holds the possible combinations of numbers of storeys and time periods of construction *still to be distributed*.
11. *Assignment of time periods of construction and numbers of storeys to buildings in subset (d):* Each of the buildings in subset (d) is randomly assigned a time period of construction and number of storeys from *what is left* in matrix K after step (10). As in step (10), each time a building is “assigned”, it is eliminated from the matrix. At any particular time, matrix K holds the same number of buildings that have not been assigned yet.

Once each building has a time period of construction, they are assigned the corresponding distribution of vulnerability classes as per Table 1.

The possible combinations of random assignments of time period of construction and/or number of storeys described in steps (10) and (11) is large. The relevance of assigning a particular combination of time period of construction and number of storeys to each building stems from the spatial variability of ground motions and, therefore, intensities, as different locations of these buildings can lead to different kinds of damage. For this reason, we used Monte Carlo sampling to generate 10 sets of 200 realisations of steps (10) and (11) each (i.e., 2000 realisations in total). Here, the order in which the buildings were assigned time periods of construction and/or number of storeys (i.e., the “list” of buildings to assign) was randomised as well, and the probability of occurrence of each damage grade was calculated. Each of these 2000 realisations led to a damage calculation that resulted in each building from subsets (c) and (d) being assigned probabilities of each damage grade from 0 through 5 occurring. As buildings in subsets (a) and (b) are always the same (i.e. their vulnerability does not depend on the steps followed to assign time periods of construction and/or numbers of storeys to the rest), their probabilities of suffering from each damage grade were calculated only once.

Strictly speaking, it was not necessary to assign numbers of storeys to buildings for which this information was missing, as it would have been possible to use the fragility model of Raschke (2003) using the vulnerability index corresponding to the case agnostic to the number of storeys displayed in Table 2. However, using the number of storeys was preferred herein because it would result in a more accurate estimate of fragility, the number of storeys was known for 96.6% of the buildings, and was needed in any case to avoid assigning unrealistic time periods of construction.

#### Final building-by-building model

The enrichment of the building-by-building CBE19 model as per the algorithm described above led to a final model in which all buildings were assigned time periods of construction and numbers of storeys. While the specific time periods of construction assigned to each building of subsets (c) and (d) change in each Monte Carlo realisation, the overall number of buildings in each category of time period of construction remains the same. It is thus possible to characterise the distribution of all residential buildings across ranges of time periods of construction and number of storeys because the random assignment was done based on a theoretical distribution. Table 7 shows the resulting classification and the resulting distribution of number of storeys per range of year of construction (percentages in parentheses), respectively. As can be observed, the percentage of buildings of different number of storeys for each time period of construction (shown in parentheses in Table 7) are consistent with the adopted theoretical distribution of Table 6. Differences are due to the adjustment of the theoretical distribution to accommodate buildings for which the number of storeys is known but not the year of construction (subset c) as well as the assumptions regarding buildings with no year of construction data having been built after 1990 (with the limitations and constraints discussed earlier).

**Table 7:** Final number of buildings per time period of construction and number of storeys for the total 169,471 residential buildings in the city of Cologne

Time period of construction	Number of storeys									
	1	2	3	4	5	6	7	8	9	≥ 10
Before 1918	2022 (14.58%)	4140 (29.85%)	3578 (25.79%)	3068 (22.12%)	950 (6.85%)	97 (0.70%)	14 (0.10%)	1 (0.01%)	0 (0.00%)	1 (0.01%)
1919–1948	5099 (23.82%)	10,777 (50.36%)	2719 (12.70%)	2177 (10.17%)	490 (2.29%)	103 (0.48%)	36 (0.17%)	1 (0.00%)	0 (0.00%)	0 (0.00%)
1949–1962	7468 (21.29%)	14,633 (41.71%)	5562 (15.85%)	5001 (14.25%)	1705 (4.86%)	446 (1.27%)	145 (0.41%)	60 (0.17%)	46 (0.13%)	19 (0.05%)
1963–1975	6368 (21.82%)	13,524 (46.33%)	3652 (12.51%)	2996 (10.26%)	1184 (4.06%)	595 (2.04%)	243 (0.83%)	294 (1.01%)	144 (0.49%)	189 (0.65%)
1976–1989	4763 (28.46%)	8515 (50.88%)	1436 (8.58%)	1116 (6.67%)	543 (3.24%)	204 (1.22%)	76 (0.45%)	24 (0.14%)	14 (0.08%)	44 (0.26%)
After 1990	18,976 (35.68%)	21,324 (40.09%)	6200 (11.66%)	4394 (8.26%)	1674 (3.15%)	441 (0.83%)	125 (0.24%)	36 (0.07%)	3 (0.01%)	16 (0.03%)

The percentages are calculated per row (i.e. distribution of number of storeys for each time period of construction)

**Fig. 9:**
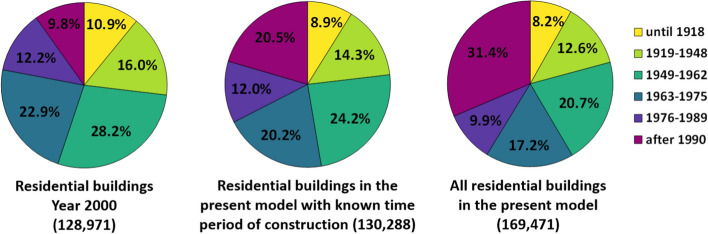
Proportion of residential buildings assigned to each time period of construction: aggregated statistics for the year 2000 (Schwarz and Maiwald 2019) (left), the present model, only buildings with time period of construction known from ODK (centre), the present model, all residential buildings (right)

The proportions of residential buildings allocated to each range of years of construction are depicted in Fig. 9 for the situation in the year 2000 (aggregated statistics from the survey by the city of Cologne; left), for buildings for which the year of construction was known from the ODK dataset (centre) and for all buildings (right). The latter is the result of all the procedures and assumptions applied herein for enriching the building-by-building model with aggregated statistics. While the accuracy of the final proportions cannot be fully assessed, the increase in the proportion of buildings built after 1990 from left to right is consistent both with the passage of time and the assumptions adopted herein for buildings for which the year of construction was not known. The distribution of years of construction among buildings built before 1990 (i.e., removing those built after 1990 from the set) is reasonably stable in the three cases. Moreover, the relatively low proportion of pre-1945 buildings in all three plots is in agreement with the fact that the city of Cologne was heavily damaged during World War II (e.g., Grünthal et al. 2006). These observations serve as consistency checks that show, for example, that the inaccuracies that may stem from potential misclassifications in Table 5 do not lead to unreasonable outcomes.

**Fig. 10:**
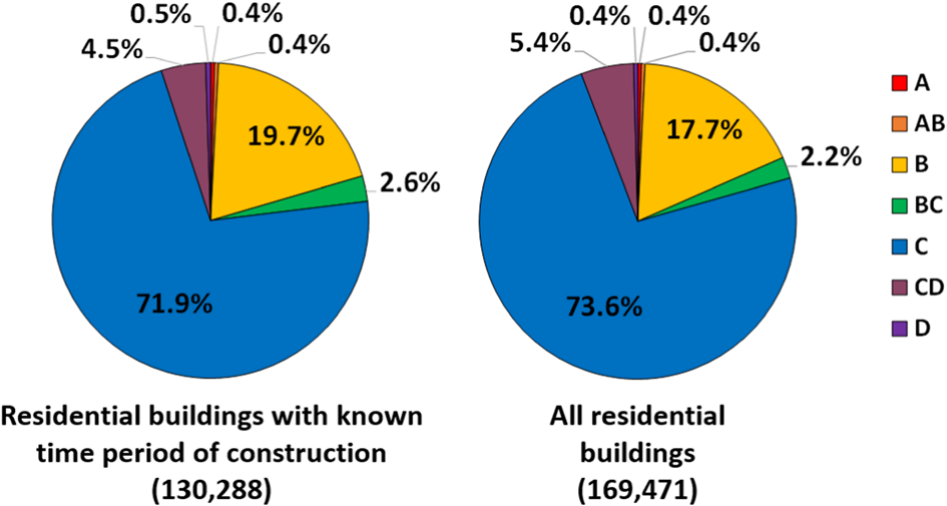
Aggregated proportion of residential buildings assigned to each vulnerability class: only buildings with time period of construction known from ODK (left), all residential buildings (right)

The overall aggregated distribution of vulnerability classes of the residential building stock of the city of Cologne can be calculated by combining the total number of buildings per range of year of construction (Fig. 9) with the distribution of vulnerability classes per range of year of construction (Table 1). The plots in Fig. 10 show the results for the buildings for which the year of construction is known on the left and all residential buildings on the right. Vulnerability class C is predominant in both cases and represents over 70% of the building stock. Around 20% of the buildings are classified as B, and less than 1% as the most fragile classes A and AB.

**Fig. 11:**
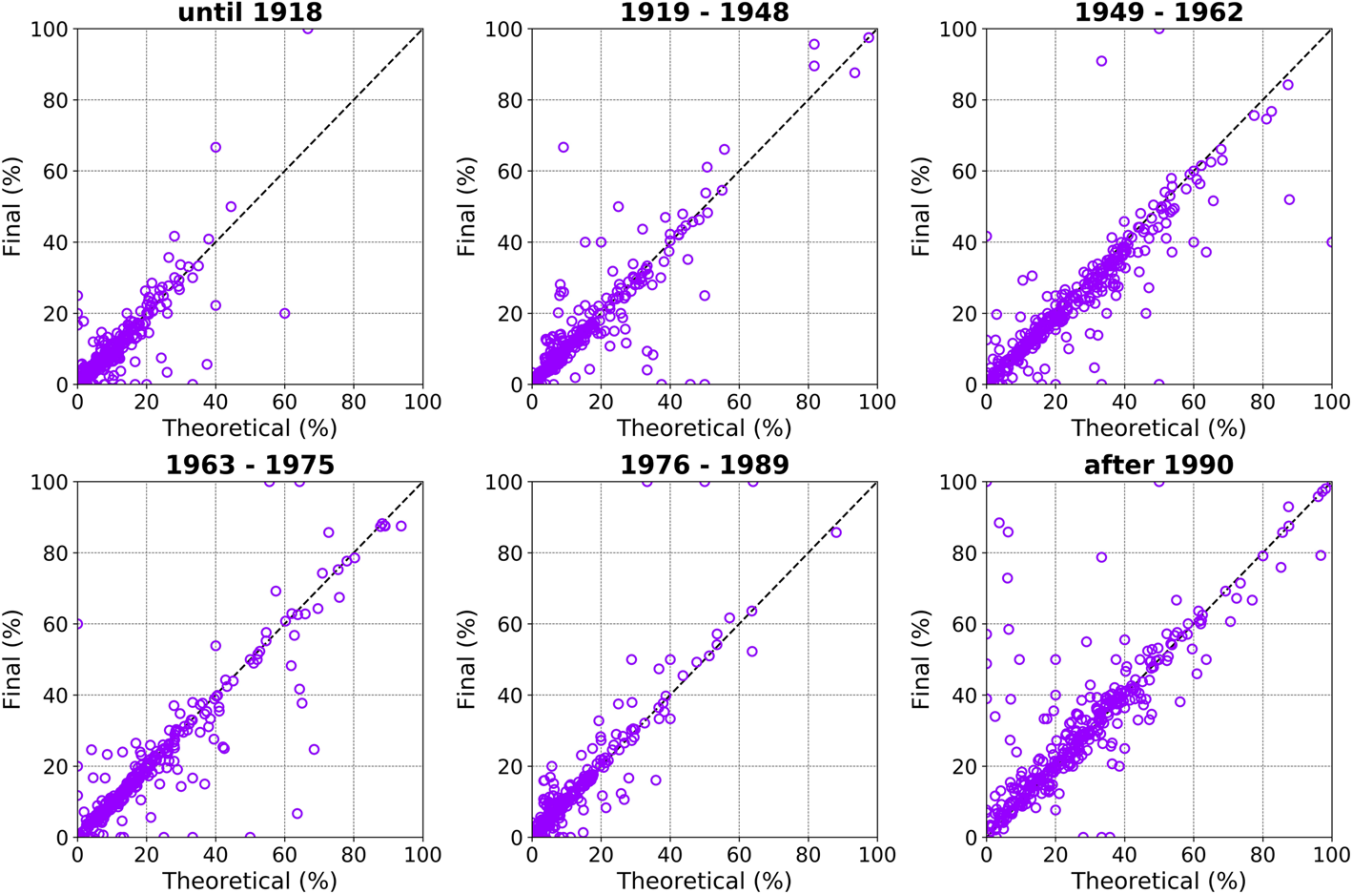
Final versus theoretical proportion of each time period of construction for the whole of Cologne. Each data point corresponds to a neighbourhood (*Viertel*, in German)

With the purpose of assessing how much the theoretical distributions of time periods of construction per neighbourhood (see Fig. 4) were preserved after the adjustments described above, Fig. 11 presents a comparison between the theoretical and the final proportions for each of the six ranges, with each point corresponding to one neighbourhood. While the points lie reasonably well-aligned with the 1:1 diagonal, the differences between the two distributions are due to the need to adjust the theoretical values to fit the observations from the building-by-building exposure model, as described in steps 6 and 8 in the previous section.

**Fig. 12:**
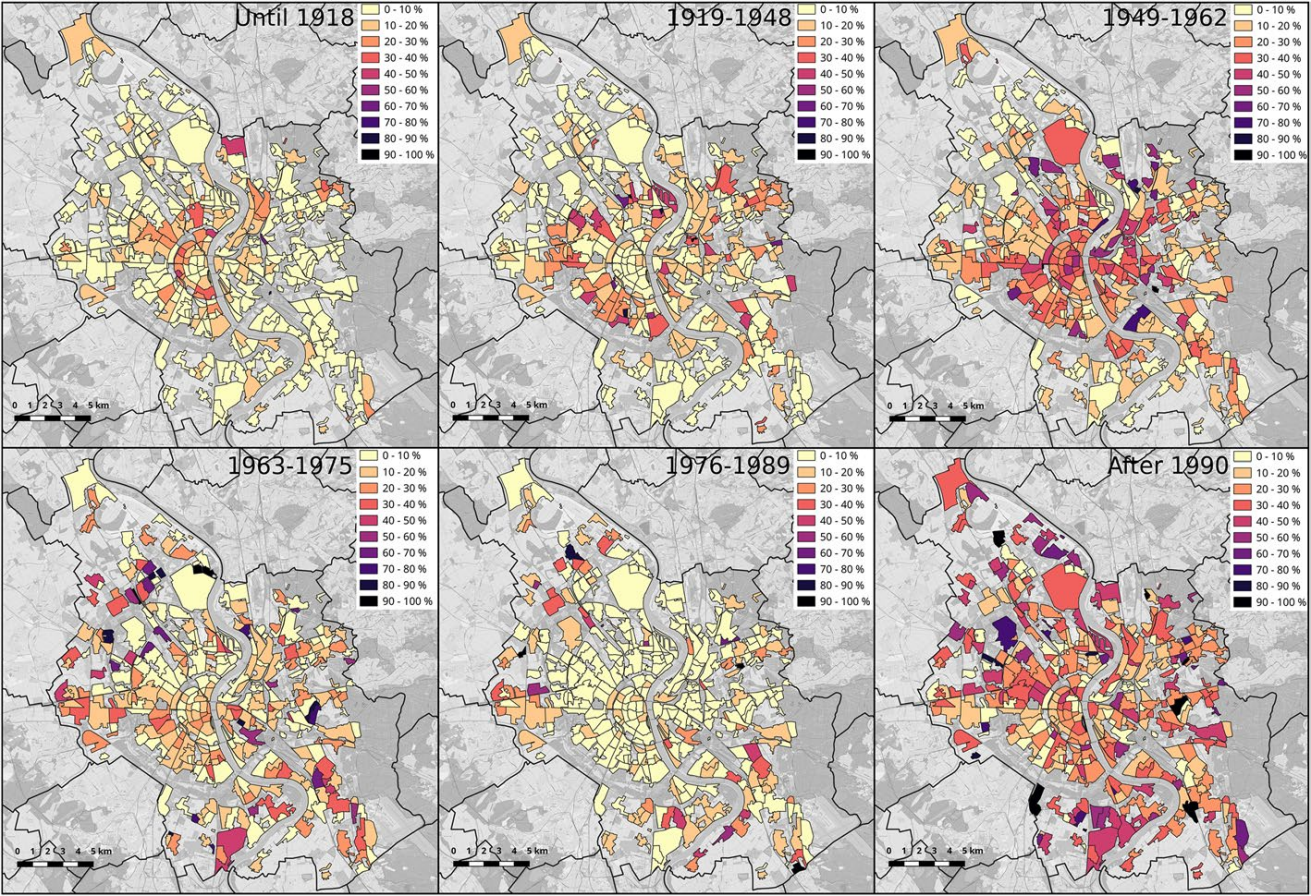
Final proportion of buildings built in each range of years of construction per neighbourhood (*Viertel*, in German) of the city of Cologne

The maps in Fig. 12 aid in visualising the spatial distribution of ranges of time period of construction by depicting the final proportion of each range per neighbourhood. The intensity reconstruction in the post-war period (1949–1962) as well as the expansion in time from the central area to the outskirts of the city are quite noticeable in the series of maps.

## Application: earthquake scenario damage calculation

### Seismological aspects of the earthquake scenario

The city of Cologne is located at the south-eastern edge of the Lower Rhine Embayment (LRE). The LRE is an active intraplate rift system whose moderate seismicity makes it one of the most active areas in Europe north of the Alps, despite its faults being characterised by relatively low slip rates of less than 0.1 mm per year (Vanneste et al. 2013). Though infrequent, there are records of damaging historical earthquakes in the area, such as the *M*_w_ 5.8 Düren earthquake of 1756 (Meidow 1995) and the *M*_w_ 6.0–6.25 earthquake of 1692 in eastern Belgium (Alexandre et al. 2008) (magnitudes estimated from intensity in both cases); even the more recent 1992 *M*_w_ 5.4 Roermond earthquake caused six finials of the Cologne cathedral (around 70 km away) to break out of their embeddings, with one causing further damage to the building during its fall (Meidow and Ahorner 1994).

The NW–SE trending Erft normal fault is one of the most prominent fault zones of the LRE and separates the Erft block to the west from the Cologne block to the east. This complex system extends for around 50 km, consists of several sections of around 10–15 km in length, and exhibits an average geological slip rate of 0.05 to 0.1 mm per year (Ahorner 1962, 1983). While Vanneste et al. (2013) assign a maximum moment magnitude *M*_w_ of 7.1 to the Erft fault, disaggregation of the seismic hazard in the city of Cologne for a peak ground acceleration (PGA) with a 475-year return period according to the 2016 national seismic hazard assessment for Germany (Grünthal et al. 2018) indicates the largest contribution stemming from *M*_w_ in the range 4.5 to 6.0 at distances of 10 to 40 km, and a minor participation of earthquakes with *M*_w_ 6.5 and above at distances up to 110 km. In view of these considerations, a scenario earthquake on the Erft fault with *M*_w_ 6.5 was selected for this study. According to the 2016 national seismic hazard assessment for Germany (Grünthal et al. 2018), a *M*_w_ 6.5 would be exceeded on the Erft fault with a return period of around 4700 years and around 2000 years within the Lower Rhine Embayment as a whole.

We defined the rupture plane by considering the geometrical definition of the Erft fault as in the 2016 national German hazard model (strike 147°, dip 57.5°, rake −87°; Grünthal et al. 2018) and all possible rectangular ruptures on the fault with an aspect ratio of 1.5 and area compatible with a *M*_w_ 6.5 earthquake according to the relationships of Wells and Coppersmith (1994). The final rupture was selected assuming a nucleation point toward the centre of the fault (epicentre located at 50.79°N, 6.74°E) with bilateral propagation, and a depth to the top of the rupture of 4 km, which leads to a hypocentral depth of around 10 km, consistent with previous seismicity on the Erft fault (Hinzen 2003). This resulted in a 20-km long rupture, with a Joyner-Boore distance to the centre of Cologne of around 18 km.

### Ground motion and intensity fields

Peak ground motion accelerations (PGA) due to the selected scenario earthquake were computed as described by Pilz et al. (2020a) by first calculating full response spectra for rock conditions (average S-wave velocity in the uppermost 30 m, *v*_S30_ = 760 m/s) and then applying a Random Vibration Theory (RVT) approach to amplify the ground motions by means of a 1D wave propagation analysis based on a detailed 3D velocity and attenuation model of the sedimentary layers of the LRE. The ground motions on rock were calculated using the ground motion model (GMM) of Boore et al. (2014), as Pilz et al. (2020a) found this model to show the lowest bias with respect to ground motions from 16 weak and moderate earthquakes (with magnitudes 3.1–4.4) recorded in and around the LRE since 2007.

Given that the fragility models used in this study are defined in terms of macroseismic intensities in the European Macroseismic Scale EMS-98 (Grünthal 1998), we converted the median PGA values and their associated dispersions into EMS-98 intensities using the ground motion-to-intensity conversion equation of Faenza and Michelini (2010). For the current purpose, we assumed direct equivalence between the Mercalli-Cancani-Sieberg (MCS) scale employed by Faenza and Michelini (2010) and the EMS-98 scale, following Musson et al. (2010). The resulting standard deviation of intensities accounts only for the uncertainty in the PGA on rock plus the uncertainty resulting from the conversion of PGA into intensity, but not for the uncertainty in the site amplification.

**Fig. 13:**
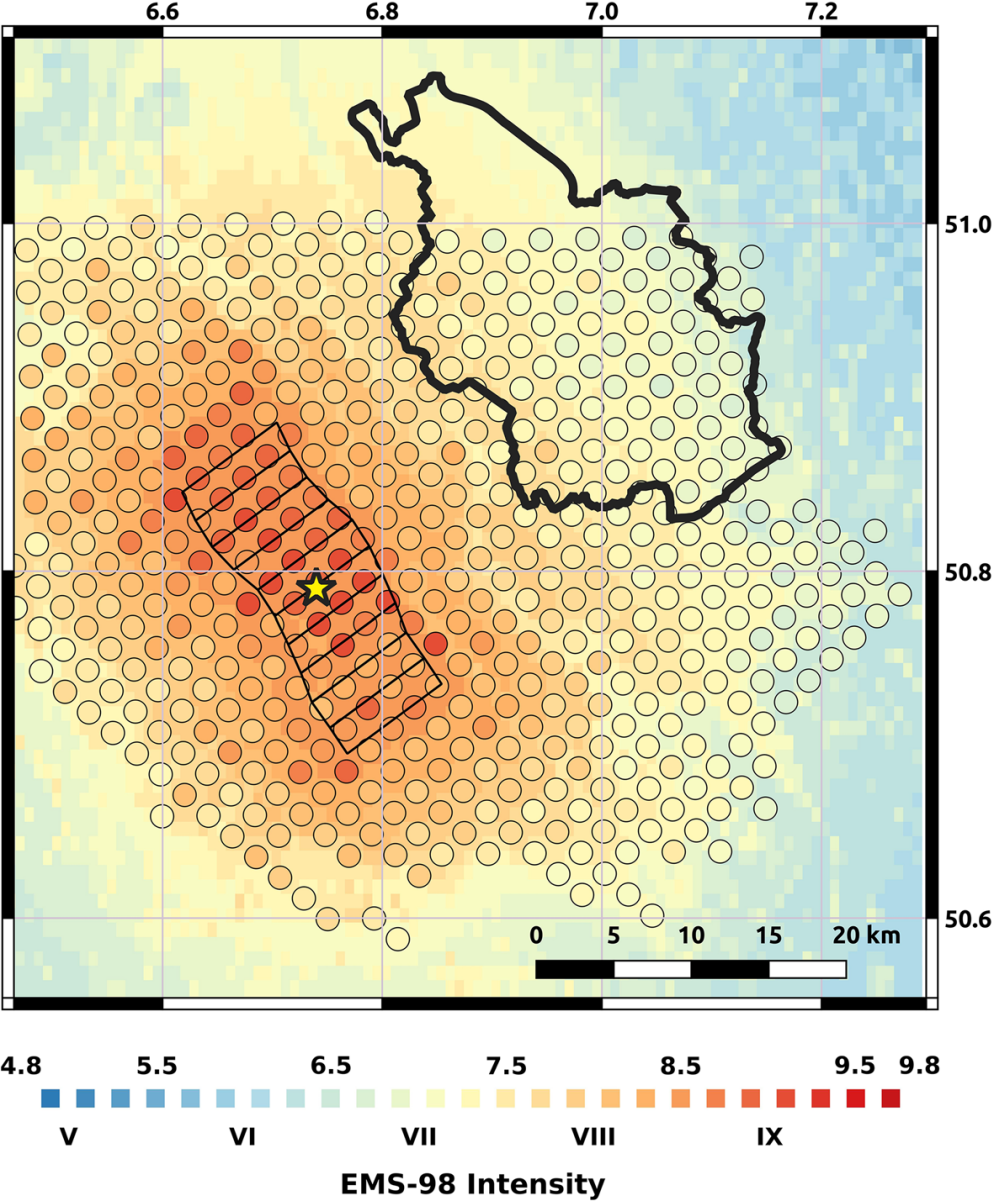
Median EMS-98 intensities at the sites where detailed soil amplification analyses were carried out by Pilz et al. (2020a) (circles) over those calculated over a 30-arcsec grid using the GMM of Boore et al. (2014) with the slope-derived proxy *v*_S30_ values of Wald and Allen (2007) (background). Polygon and star: projection of the rupture to the surface and epicentre of the scenario earthquake. Thick contour: administrative boundary of Cologne

As the detailed amplification model of Pilz et al. (2020a) does not cover all of the city of Cologne, we calculated an additional PGA field using the GMM of Boore et al. (2014) with the slope-derived proxy *v*_S30_ values of Wald and Allen (2007) over a 30-arcsec uniform grid, and depths to the 1 km/s shear velocity layer (Z_1_) estimated by means of the empirical relation of Chiou and Youngs (2014). This field was converted into EMS-98 intensity, as described before, and calibrated to align with the field resulting from the detailed amplification model. Figure 13 shows the two resulting fields of median intensities and an agreement that is overall satisfactory. The detailed model was adopted in the area where it was available, while the calibrated model was adopted in the northern part of the city, which holds approximately 12% of the total number of residential buildings. This merging procedure (details can be found in Pilz et al. 2020b) is likely to have resulted in slightly over-conservative estimates of the intensities affecting this small subset of buildings.

### Calculation of damage grades

The calculation of the probability of each specific building resulting in each particular damage grade was carried out accounting for the uncertainty in the vulnerability class, by considering the probabilities of each class VC_*k*_ associated with the corresponding time period of construction shown in Table 1. At the same time, the uncertainty in the macroseismic intensity was considered by discretising a normal distribution defined by the median intensity and its standard deviation at the site using bins of 0.5 intensity width with average EMS-98 intensity I_*j*_. The intensity field (median and standard deviation) was interpolated to retrieve the values corresponding to the centroid of each building. The building-to-building variability of buildings under the same vulnerability class was partially accounted for through the fact that the component of the fragility model of Raschke (2003) that translates the mean damage grade *D*_m_ into a distribution of discrete damage grades was derived using empirical earthquake damage data. These data can be expected to include buildings with different characteristics within each individual vulnerability class, though it is not known how well sampled each class is. However, each combination of vulnerability class and number of storeys was still represented by a “fixed” set of fragility curves representing a central tendency. The use of more sophisticated analytical approaches (e.g. Gentile and Galasso 2020) could be a development for future applications.

The probability of observing damage grade *i* was thus calculated as per Eq. (1):

1 $$P\left[ {DG_{i} } \right] = \mathop \sum \limits_{j} \mathop \sum \limits_{k} P\left[ {I_{j} } \right] \cdot P\left[ {VC_{k} } \right] \cdot P\left[ {\left. {DG_{i} } \right|I_{j} ,VC_{k} ,n_{s} } \right]$$

In Eq. (1), *P*[*DG*_*i*_|*I*_*j*_, VC_*k*_, *n*_s_] is the probability of a building with vulnerability class *k* and number of storeys *n*_s_ subject to EMS-98 intensity *I*_*j*_ suffering damage grade *i*, and is the equivalent of P[DG_*i*_ |*I*, *C*] in Eq. (6), as the vulnerability index *C* depends on VC_*k*_ and *n*_s_.

### Damage results

One of the advantages of using a building-by-building exposure model is to be able to determine the number or proportion of buildings expected to be exposed to a certain ground motion level eliminating the uncertainty in the spatial distribution of the buildings that otherwise exists in aggregated models. For the earthquake scenario considered herein, 70.02% of the residential buildings are expected to be located in areas with resulting EMS-98 macroseismic intensity VII (defined numerically as 6.75–7.25), while 29.55% are expected to be located in the transition between VII and VIII (defined numerically as 7.25–7.75). Only 0.38% and 0.04% are expected to be located in areas with intensity VI–VII and VIII, respectively. EMS-98 intensity of VII is described as “damaging” shaking, with many buildings of vulnerability class A suffering damage of grade 3 (moderate structural and heavy non-structural damage) and a few of grade 4 (heavy structural and very heavy non-structural damage), many buildings of vulnerability class B suffering damage of grade 2 (slight structural and moderate non-structural damage) and a few of grade 3, a few buildings of vulnerability class C sustaining damage of grade 2, and a few buildings of vulnerability class D sustaining damage of grade 1 (no structural and slight non-structural damage) (Grünthal 1998).

The damage calculations result in each building being assigned a probability of suffering from damage grades 0 through 5 when subject to the scenario earthquake considered herein. This was done just once for buildings in subsets (a) and (b) but 2000 times for all other buildings. For the latter, mean results across all 200 realisations of each of the 10 sets were compared with one another and against mean results from all 2000 realisations considered together, so as to gauge the stability of the Monte Carlo simulations with respect to the set size. Results that aggregate numbers of buildings for the whole city were very stable across all realisations and sets, and the variability was very small, even when looking only at buildings from subsets (c) and (d). The mean across all 2000 realisations is thus presented in what follows. However, the impact of the Monte Carlo simulations on the variability of the calculated damage can be observed when analysing results for individual buildings, as will be shown shortly.

Table 8 shows the number and proportion of buildings that result with a certain probability of suffering from each damage grade. The summation of each row is equal to the total number of residential buildings and 100%, respectively, because all buildings have some probability of suffering from each damage grade. More than half of the total number of residential buildings have a 20–30% chance of suffering from DG1 (no structural and slight non-structural damage), while a further 39% have a larger chance of 30–40%. In turn, around half of the buildings have less than a 10% chance of suffering from DG2 (slight structural and moderate non-structural damage), while a further 33% and 16% have 10–20% and 20–30% probability of the same damage grade. The proportion of buildings with less than 10% chance of observing a specific damage grade increases drastically for DG3 and above, although around 14% of the buildings have at least a 10% chance of resulting in DG3. Almost all buildings resulted in less than 5% chance of suffering from very heavy structural and non-structural damage (DG5). The proportion of buildings allocated to each probability range of suffering no damage (DG0) is informative too. The fact that very small numbers of buildings lie in either of the extremes (probabilities smaller than 20% or higher than 80%) conveys that the scenario earthquake may be expected to be neither largely destructive nor innocuous. Table 8 suggests that light to moderate damage (DG1 and DG2) could be widespread. While likely repairable, the economic costs of such degrees of damage could be considerable.

**Table 8:** Number and percentage (in parentheses) of residential buildings classified according to their probability of observing each damage grade

Damage grade	Probability of occurrence (%)									
	0–10	10–20	20–30	30–40	40–50	50–60	60–70	70–80	80–90	90–100
DG0	1571 (0.93%)	7417 (4.38%)	10,382 (6.13%)	15,766 (9.30%)	22,642 (13.36%)	36,301 (21.42%)	40,727 (24.03%)	29,829 (17.60%)	4836 (2.85%)	0 (0.00%)
DG1	9 (0.01%)	16,032 (9.46%)	87,827 (51.82%)	65,603 (38.71%)	0 (0.00%)	0 (0.00%)	0 (0.00%)	0 (0.00%)	0 (0.00%)	0 (0.00%)
DG2	86,340 (50.95%)	56,657 (33.43%)	26,474 (15.62%)	0 (0.00%)	0 (0.00%)	0 (0.00%)	0 (0.00%)	0 (0.00%)	0 (0.00%)	0 (0.00%)
DG3	145,950 (86.12%)	17,086 (10.08%)	6435 (3.80%)	0 (0.00%)	0 (0.00%)	0 (0.00%)	0 (0.00%)	0 (0.00%)	0 (0.00%)	0 (0.00%)
DG4	163,380 (96.41%)	5697 (3.36%)	387 (0.23%)	7 (0.00%)	0 (0.00%)	0 (0.00%)	0 (0.00%)	0 (0.00%)	0 (0.00%)	0 (0.00%)
DG5	169,415 (99.97%)	56 (0.03%)	0 (0.00%)	0 (0.00%)	0 (0.00%)	0 (0.00%)	0 (0.00%)	0 (0.00%)	0 (0.00%)	0 (0.00%)

The same conclusion can be reached when considering results in an aggregated fashion, by treating the probabilities of each building suffering from a specific damage grade as fractions of buildings that can be added up in a statistical sense. The results of such an aggregation process are presented in Table 9, both in terms of occurrence and exceedance. According to these, around half of the residential buildings in Cologne are not expected to suffer any damage, while slightly over a quarter are expected to suffer from DG1, that is, from only slight non-structural damage, and a 12% are expected to suffer from DG2, i.e., slight structural and moderate non-structural damage. Although the number of buildings expected to suffer from DG4 and DG5 represent a small fraction of the building stock, the potential consequences stemming from these buildings should not be underestimated, particularly as they may result in severe injuries and deaths. However, it is also noted that DG5 includes a range of extents of collapse and very heavy structural damage and cannot be fully equated to complete flattening of the structures in all instances.

**Table 9:** Expected numbers and proportions of residential buildings experiencing and exceeding different damage grades, obtained by treating results in an aggregated manner

Damage grade	Occurrence		Exceedance	
	Number	%	Number	%
DG0	92,328.6	54.5	169,471.0	100.0
DG1	46,355.4	27.4	77,142.4	45.5
DG2	19,703.8	11.6	30,787.0	18.2
DG3	7947.7	4.7	11,083.2	6.5
DG4	2691.5	1.6	3135.6	1.9
DG5	444.1	0.3	444.1	0.3

A simplified calculation was carried out with the purpose of analysing the similarity of these results to those stemming from an aggregated exposure model for the whole city. The final distribution of all residential buildings per number of storeys and time period of construction (Table 7) was combined with the probability of each time period of construction being associated with a specific vulnerability class (Table 1) to obtain the distribution of all residential buildings per number of storeys and vulnerability class. The expected damage grades were calculated with the latter and the median EMS-98 intensity (7.13) and standard deviation (0.7) for all buildings. The resulting aggregated exposure model inherently contains building-level information, as Table 7 results from the process of combining datasets with different spatial and temporal resolutions described in this paper (Fig. 4 and steps 6 and 8 in Fig. 5). Results obtained by means of this simplified aggregated calculation were very similar to those shown in Table 9. However, the good agreement obtained herein cannot necessarily be generalised. The extent to which different resolutions of exposure lead to similar or dissimilar results depends on the scenario (proximity to the fault, spatial variability of site effects, resolution at which site characterisation is available) and the nature of the peril (e.g., Gomez-Zapata et al. 2021).

**Fig. 14:**
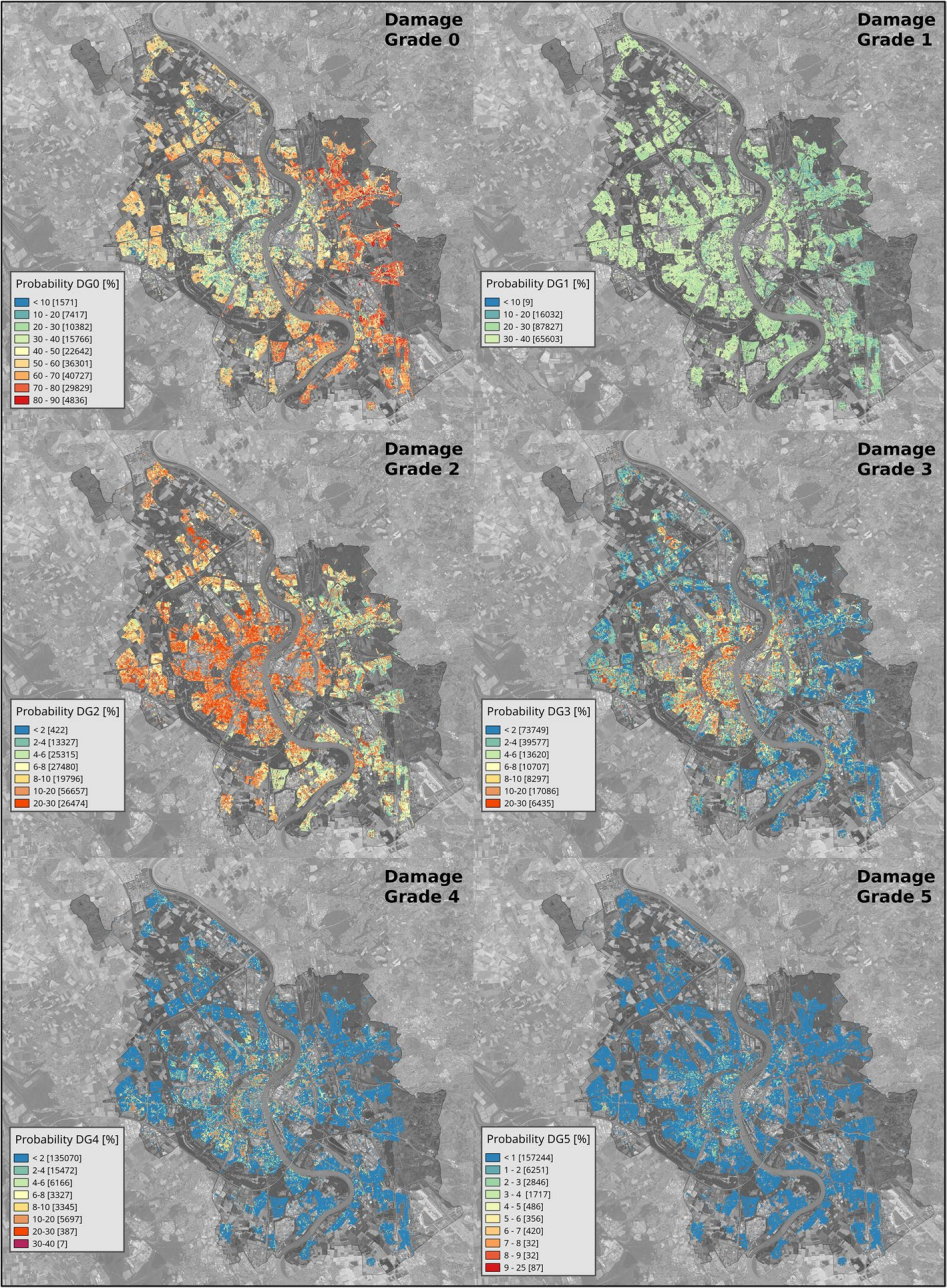
Probability of occurrence of each EMS-98 damage grade for each residential building. Background orthophotos from Bezirksregierung Köln (2021)

Even if results obtained using a simplified aggregated version of the exposure model are quite similar (in an aggregated sense), carrying out calculations at the building-by-building level enables the investigation of the spatial distribution of the expected resulting damage, which can be better observed in the maps in Fig. 14, where the probabilities of observing each damage grade are depicted for each individual building. From these maps it appears that the more extreme cases of damage are more likely to occur around the central area, west of the river, and further west. This is consistent with both the location of the causative fault (Fig. 13) and the spatial distribution of years of construction per neighbourhood (Fig. 12). There is a correspondence, for example, between some of the neighbourhoods shown to comprise the larger proportions of buildings built before 1918 in Fig. 12 and the areas with the larger probabilities of suffering from damage grades 2, 3 and 4 in Fig. 14. Similarly, the lower degrees of damage expected on the eastern sector of the city are in agreement with the larger proportions of more modern buildings shown for these neighbourhoods in Fig. 12, as well as the lower intensities in this area (Fig. 13). The spot that shows higher probabilities of damage grades 2 and 3 in the northern parts of the city is likely due to a combination of a higher proportion of 1963–1975 buildings (compared to other neighbourhoods around the same area) and a slight overestimation of the ground motion intensities in the north due to the merging of the two intensity fields.

**Fig. 15:**
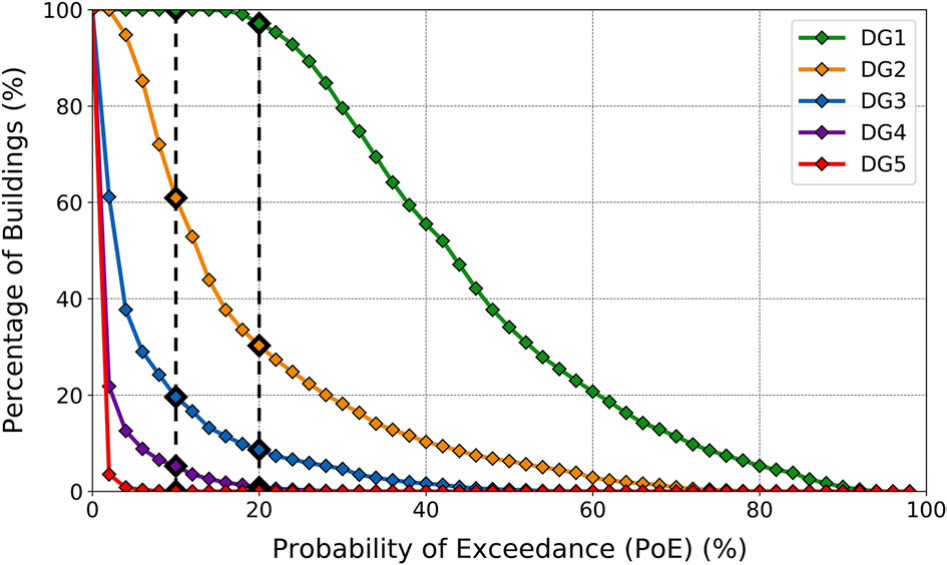
Percentage of buildings with probability of exceedance (PoE) of each EMS-98 damage grade (colour scale) equal to or greater than indicated in the horizontal axis. The vertical dashed lines correspond to the examples given in the text

Figure 15 depicts an alternative representation of the results by showing the percentage of residential buildings with probability of exceedance of each damage grade equal to or larger than the values plotted along the horizontal axis. From this figure it can be observed, for example, that all buildings have at least a 10% probability of exceeding DG1, while for the rest of the damage grades the percentages of buildings are 60.9% (DG2), 19.6% (DG3), 5.3% (DG4) and 0.03% (DG5). When looking at a 20% probability of exceedance these percentages of buildings drop to 97.1% (DG1), 30.3% (DG2), 8.7% (DG3), 0.8% (DG4) and 0% (DG5). From these numbers and Fig. 15 in general it is concluded that the possibility of observing very heavy damage (DG4-DG5) cannot be ruled out, and the possibility of observing substantial to heavy damage (DG3) is not negligible at all.

**Fig. 16:**
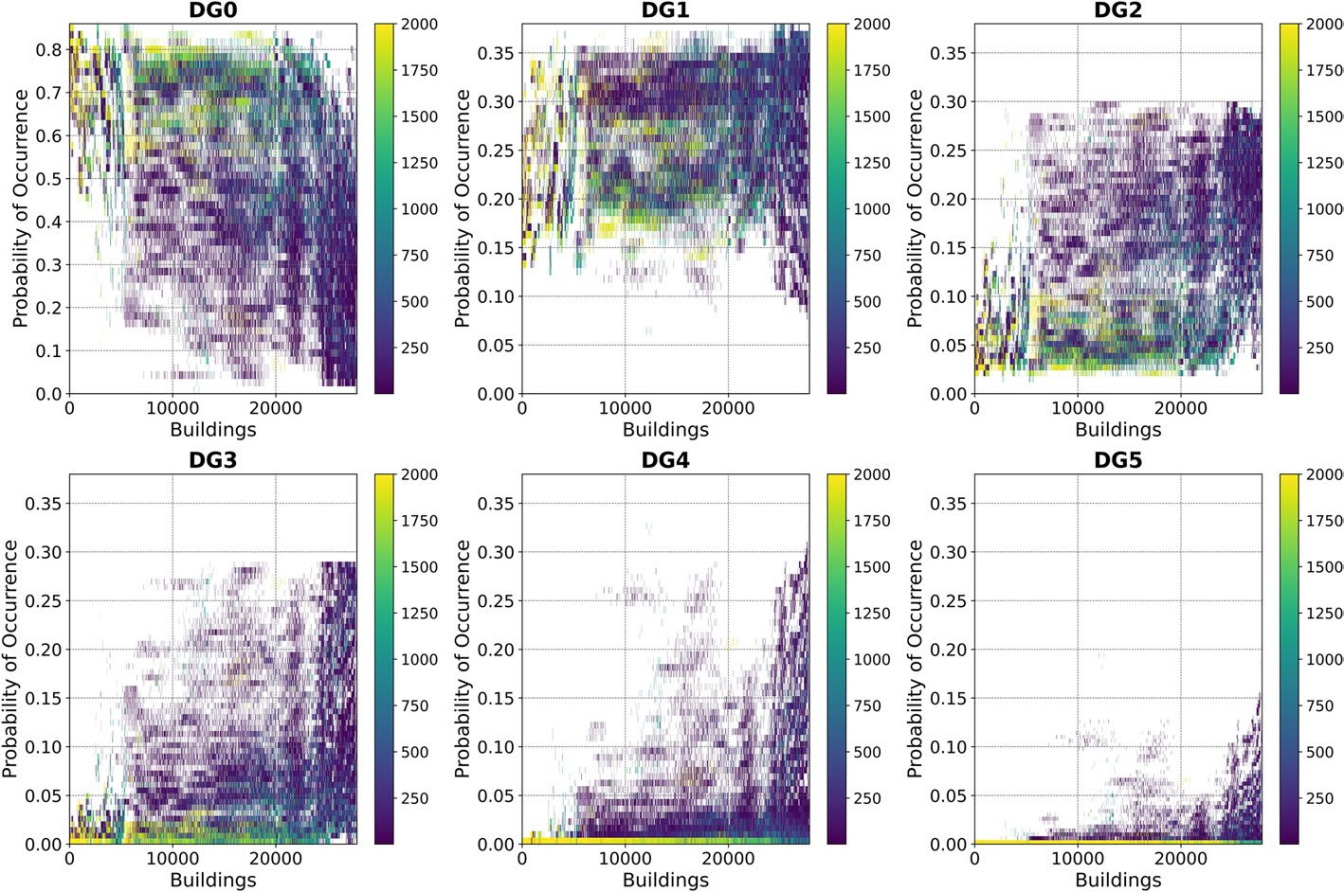
Probability of occurrence of each damage grade for each individual building, ordered from buildings with the smallest to the largest variability (only buildings with variability shown, see Appendix D for details). The colour scale indicates the number of realisations that yielded each value of the probability. Note that the plot for DG0 uses a different scale for the vertical axis from that of the other plots

**Fig. 17:**
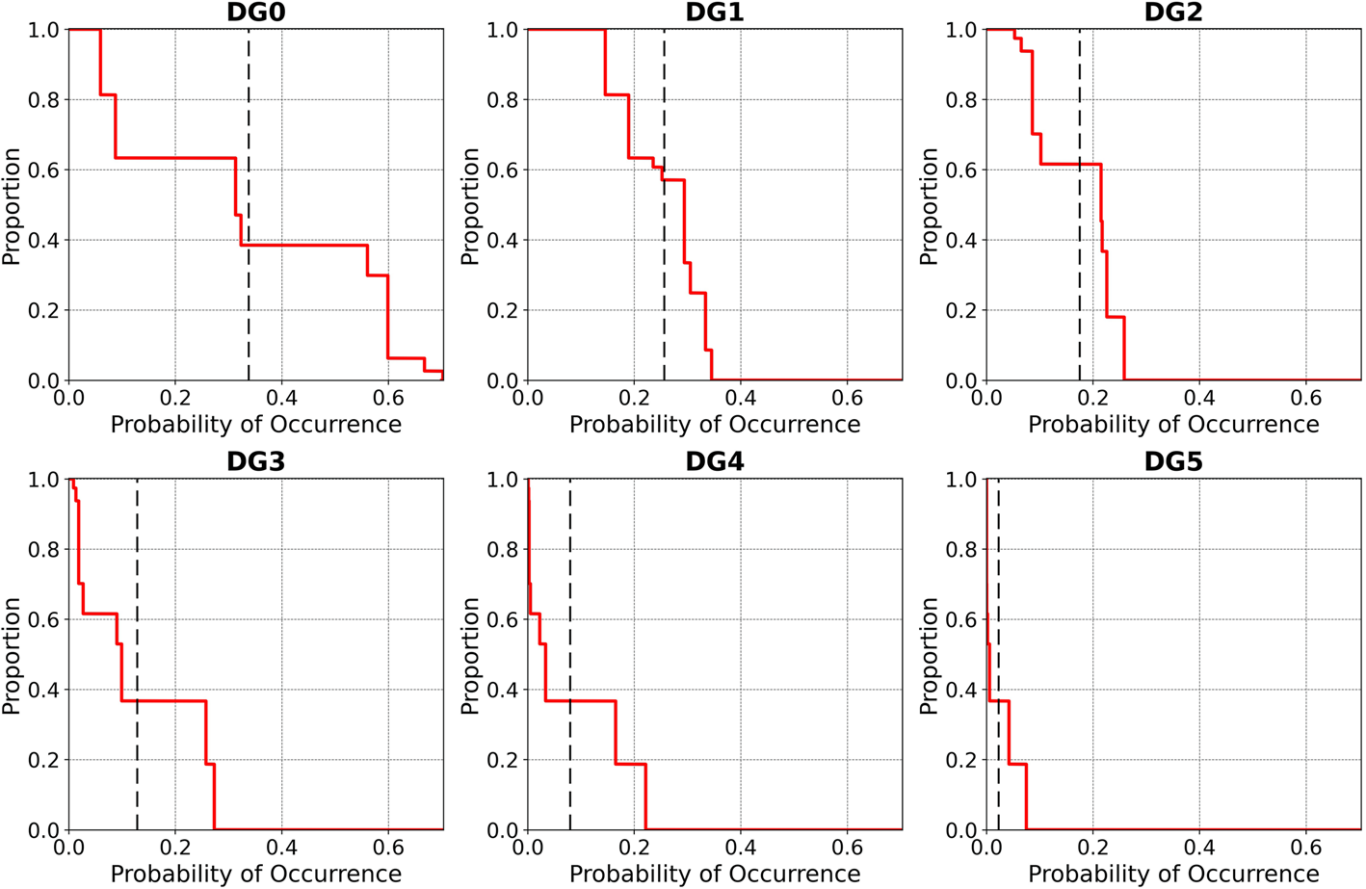
Proportion of the total 2,000 realisations for which each probability of occurrence was exceeded (red lines) and mean value of all realisations (dashed black lines) for one particular building selected as a case-study

While the results shown above proved to have very little variability when comparing the 10 sets of 200 realisations each against each other, the impact of the uncertainty in the time period of construction and/or number of storeys of the buildings for which these properties were not known in CBE19 is visible at the building-by-building level. Figure 16 depicts the density plots of the probabilities of occurrence of each damage grade for each building for which the variability is relevant, with the buildings ordered in the horizontal axis with increasing degrees of variability (see Appendix D for details). As an example, Fig. 17 focuses on one building and shows the proportion of the total number of realisations for which each probability level is exceeded (red lines), as well as the mean value of all realisations (dashed black line). As can be observed from both plots, each realisation can yield quite different probabilities of a building suffering from any particular damage grade.

### Comparison with observations following the 1978 Albstadt earthquake

The validation of results from damage and risk assessments is generally challenging, particularly so in areas of low-to-moderate seismicity, due to the lack of sufficient data from past similar earthquakes and/or to them having occurred too far away in time for the building stocks and their vulnerability to be comparable to the present day exposure. For the Cologne case, it is interesting to note that the 1999 *M*_*w*_ 6.0 Athens earthquake, which occurred at a distance from Athens comparable to that of the present scenario, caused damage to at least 53,000 buildings, while the 2011 *M*_*w*_ 6.1 Christchurch earthquake, which occurred at a shorter distance, caused damage to around 100,000 buildings, though many had already been weakened by the previous September *M*_*w*_ 7.0 event (NOAA Significant Earthquake Database, NGDC). The over 77,000 residential buildings (plus an unknown number of those of other occupancy kinds) expected to be affected to a greater or lesser extent in Cologne according to our study lie comfortably between these values. However, many of the Christchurch buildings were affected by widespread liquefaction that resulted in foundation settlements and movements, structural tilting/distortions and the failure of bearings (Cubrinovski et al. 2011), phenomena that were not modelled in the present study. Moreover, there are significant differences in seismic vulnerability between Cologne and Christchurch, as seismic design has been present in New Zealand building codes since 1939, from much longer than in Germany, and timber frame construction makes up a larger proportion of the building stock (Uma et al. 2013). While a degree of validation could only really be achieved if a similar earthquake had recently occurred in the Cologne region itself (and even so, there would be the limitations of all the uncertainties involved), looking at other past events that are likely to have caused similar ground motions and affected similar kinds of constructions is relevant as a plausibility check for the models developed and used. In this spirit, the *M*_*w*_ 5.2 earthquake that occurred close to the city of Albstadt (Baden-Württemberg, Germany) on 3 September 1978 at 05:08 UTC forms a more appropriate point of comparison for the present work, as construction practices in Albstadt and Cologne can be expected to be reasonably similar in a context where a perfect match is unfeasible.

**Fig. 18:**
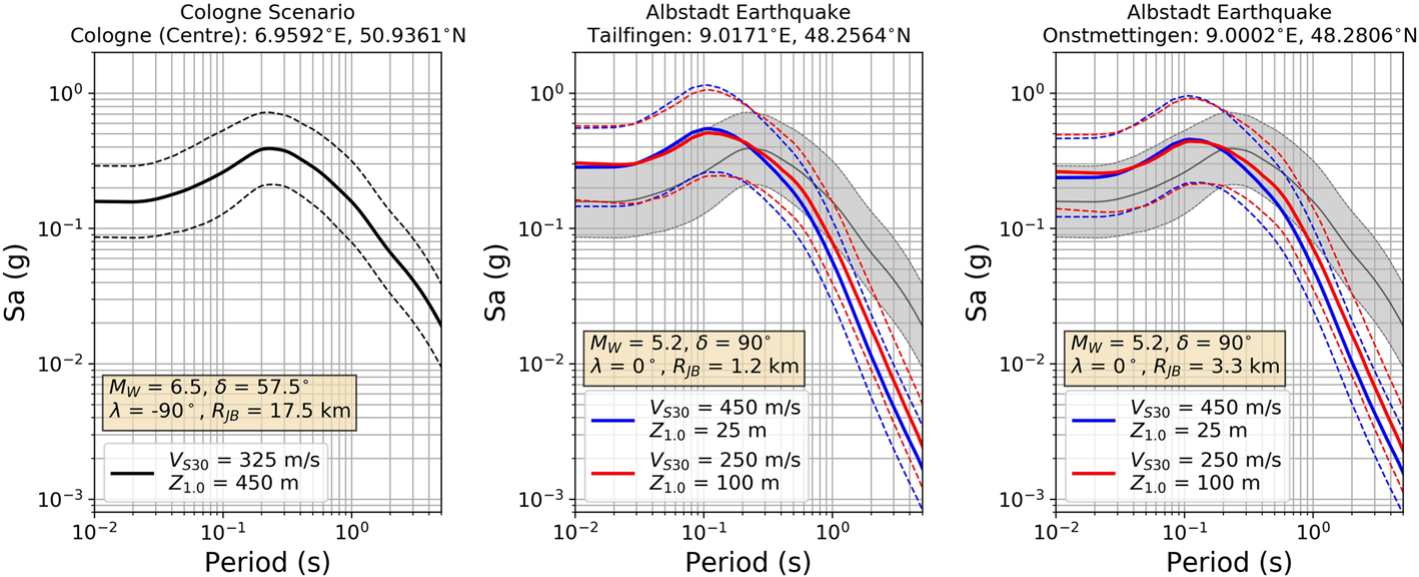
Response spectra (median ± one standard deviation) for a site in the centre of Cologne under the earthquake scenario studied herein (left) and for the centre of the Albstadt districts of Tailfingen (centre) and Onstmettingen (right) under a scenario reproducing the 1978 *M*_w_ 5.2 Albstadt earthquake, calculated using the Boore et al. (2014) ground motion model. The grey shading in the centre and right plots shows the median ± one standard deviation range for the case of Cologne to ease comparison

The Albstadt earthquake is often named as one of the most damaging earthquakes ever recorded in Germany. The Albstadt districts of Tailfingen, Onstmettingen and Ebingen were the most affected (Fig. 21, Appendix C). Despite it having had a much smaller magnitude than the *M*_w_ 6.5 considered herein for Cologne, its rupture is understood to have been located much closer to the affected sites (Fig. 21, Appendix C). In order to assess whether a comparison between the two earthquakes would be of relevance, the response spectra of both scenarios at the affected sites have been calculated using the Boore et al. (2014) ground motion model and values of *v*_s30_ and depth to the 1.0 km/s (Z_1_) layer deemed to be representative of the corresponding sites. Details on the configuration of the Albstadt earthquake scenario can be found in Appendix C. The selected rupture results in Joyner-Boore distances of around 1 to 3 km to Tailfingen and Onstmettingen, while the Joyner-Boore distance to the centre of Cologne is, under the scenario considered herein, around 17 km instead. As shown in Fig. 18, spectral acceleration values appear to be higher for the Albstadt case at the shorter periods and for the Cologne case at the longer periods, partly due to the contrast in sediment thickness, which is much greater for Cologne than Albstadt, with the crossing point lying at a period of around 0.2–0.3 s. Consideration of uncertainty through the ± one standard deviation range shows a significant overlap of ground motion values due to the two earthquakes at the sites of interest, particularly in the 0.1 to 0.5 s range, which is most relevant for the structural types involved. This is judged from the predominant number of storeys in the present model for Cologne, with an assumption of mostly masonry and infilled reinforced concrete structures, and the structural types reported by Beinersdorf et al. (2013) for Albstadt, in combination with the summary of relations between building height/number of storeys and fundamental period of vibration reported by Martins and Silva (2020). The median ground motions expected in Tailfingen and Onstmettingen generally lie within ± one standard deviation of those for Cologne in the range 0.1–0.5 s. It is noted that the detailed site amplification procedure of Pilz et al. (2020a) was not used herein to estimate the ground motions in Cologne for this comparison, so as to be consistent in the method used for both earthquakes.

The vulnerability of the building stock exposed to the 1978 Albstadt earthquake in the districts of Tailfingen and Onstmettingen described by Beinersdorf et al. (2013) appears to be broadly comparable with that for Cologne as per the exposure model developed herein, at least in terms of EMS-98 vulnerability classes, despite the differences in time periods of construction covered (post-1978 buildings only exist in the Cologne case). Table 10 shows the proportions of different vulnerability classes for the two buildings stocks. Columns labelled “grouped” were calculated by splitting the buildings allocated to intermediate categories like AB, BC, etc., evenly between the two categories that define them (e.g., vulnerability class BC was split into vulnerability classes B and C). The percentages of buildings in the extreme categories (A, AB, CD, D, DE, E) are low in both cases. When looking at the grouped values for classes B and C, it is clear that the Cologne building stock is less vulnerable than that of Albstadt in 1978, with a larger percentage of buildings of Cologne falling into the C class than those of Albstadt, and a larger percentage of buildings of Albstadt falling into the B class than in the case of Cologne. Focusing only on the pre-1976 Cologne buildings (rightmost columns of Table 10), the percentages of buildings in classes B and C become much more similar, suggesting that one of the main differences in the composition of the building stocks is due to the around 40% of post-1976 buildings in Cologne.

**Table 10:** Comparison between the vulnerability-class composition of the building stock exposed to the 1978 Albstadt earthquake in the districts of Tailfingen and Onstmettingen according to Beinersdorf et al. (2013) and of the building stock of Cologne in 2019 (all time periods of construction and only pre-1976 buildings)

Vulnerability class	Albstadt (Beinersdorf et al. 2013)		Cologne, CBE19, all		Cologne, CBE19, pre-1976	
	Individual (%)	Grouped (%)	Individual (%)	Grouped (%)	Individual (%)	Grouped (%)
A	0.0	0.1	0.4	0.6	0.6	0.9
AB	0.2		0.4		0.6	
B	14.7	37.8	17.7	18.9	30.1	31.6
BC	45.9		2.2		2.3	
C	31.1	54.8	73.6	77.4	64.0	66.1
CD	1.4		5.4		1.8	
D	6.3	7.1	0.4	3.1	0.5	1.4
DE	0.2		0.0		0.0	
E	0.2	0.3	0.0	0.0	0.0	0.0

**Fig. 19:**
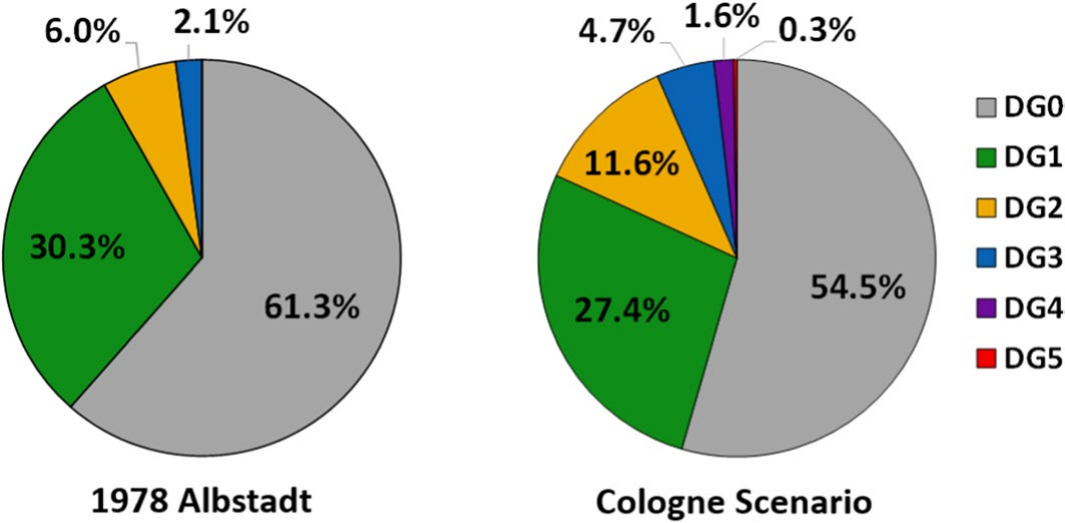
Comparison between the proportions of EMS-98 damage grades caused by the 1978 Albstadt earthquake as reported by Beinersdorf et al. (2013) for the districts of Tailfingen and Onstmettingen (left) and those calculated for the city of Cologne under the earthquake scenario studied herein (right)

All these considerations indicate that, notwithstanding the aforementioned differences and associated uncertainties, a comparison of damage from the two earthquakes may be pertinent as a point of reference for the damage analysis carried out in the present work, though it is not intended and should not be interpreted as a validation. Figure 19 shows the proportion of residential buildings of Cologne (Table 9) suffering from each EMS-98 damage grade and those reported by Beinersdorf et al. (2013), who reinterpreted the damage descriptions for the Albstadt districts of Tailfingen and Onstmettingen at the time of the earthquake (assumed to correspond mostly, if not fully, to residential buildings). It is noted that the latter do not include the district of Ebingen, which was affected as well. As can be observed, the Cologne scenario earthquake would be expected to produce greater damage than that observed for the Albstadt earthquake, though the overall consistency of proportions suggests our results for Cologne are reasonable.

## Discussion and conclusions

The procedure described herein has shown how data defined at different spatial and temporal resolution levels can be successfully combined to produce a building-by-building exposure model that is an improvement over that which would result from only considering properties and parameters available at any single resolution level alone. It is worth noting that the aggregated statistics used had their origin not only in stand-alone sources such as the distribution of ranges of time periods of construction per neighbourhood (Schwarz and Maiwald 2019) but also in the available building-by-building data, in the form of the distribution of number of storeys per range of time period of construction (Table 6). Moreover, the building-by-building years of construction were used to update the statistics at the neighbourhood level from the year 2000 to the end of 2019. Combining these sources enables the risk modelling community to benefit as much as possible from the information that each resolution level is able to provide, and to immediately incorporate new buildings and data from datasets that are updated much more frequently than censuses are carried out. Though the sort of data available for a target geographical area will dictate the specificities of the building exposure model that can be created, the main contribution of our work lies in the use of Monte Carlo simulations to fill in the gaps in information at the building-by-building level, as well as the fact that the simulations are constrained to give priority to the information coming from the available datasets and not from generic distributions. Given the nature of the problem, the approach can only be a flexible one (like the one presented herein) and needs to be adapted to the available data. If, for example, statistics on the time period of construction had not been available for the city of Cologne, statistics available at a larger geographical scale or judgement stemming from the consultation with regional experts could have been used instead.

It has also been shown that results extracted from an earthquake damage scenario calculated using a building-by-building exposure model can be presented in a variety of ways, each of which may be suited for different purposes. Tables such as Table 8 convey how the building stock as a whole is likely to be distributed with respect to the probability of suffering from each particular damage grade. For example, the fact that the proportion of buildings with less than 10% chance of observing a specific damage grade increases drastically for DG3 and above suggests that it would be rather unlikely for such a scenario earthquake to devastate the city of Cologne. At the same time, the small but non-negligible numbers of buildings that present slightly higher probabilities of suffering from the larger damage grades indicate that isolated instances of extensive damage are not unexpected either. Such a representation of results allows us to convey the probabilistic component of the analysis better than aggregated summaries like that of Table 9 (and the right plot on Fig. 19) can. The latter can, however, be more practical when communicating results with non-expert audiences and comparing against past events (as done herein with the 1978 Albstadt earthquake), though it should never be interpreted as a deterministic prognosis. One of the main advantages of working at the building-by-building level comes, however, when rendering the probabilities of each building suffering from each damage grade on a map, as in Fig. 14. While the relatively large scale of the maps shown herein does not allow for details to be identified (and this may be hardly wanted in any case due to potential privacy concerns), the colours are in fact rendered per building and a zoom in would, for example, enable post-disaster emergency agencies to identify buildings particularly at risk and respond accordingly. Further estimations of road blockages could be carried out as well to improve the response, knowing the position of the buildings and applying models of falling debris (e.g., Costa et al. 2020). Moreover, as the use of OpenStreetMap allows us to identify all kinds of buildings, it is possible to characterise the extent of the damage in buildings around, for example, relevant hospitals, to try to understand if accessibility to them has deteriorated due to the damage to neighbouring buildings or not, and to make estimates of post-earthquake business downtimes induced by restricted access due to safety cordons around buildings identified to be at risk of collapse (e.g., Hulsey et al. 2018). It should be highlighted at this point that the study carried out has focused on residential buildings and has not covered critical infrastructures, as the latter require specific knowledge on their structural characteristics, not only due to them often being fundamentally different from residential structures but also because the scale of the consequences that arise from their failure is much larger and warrants increased confidence in their characterisation.

While we have focused on presenting damage results using the mean of all 2,000 realisations, the strength of the adopted Monte-Carlo procedure resides in its capacity to produce full individual realisations of damage for the complete residential building stock. In our particular case-study the variability of results per realisation was not large when aggregating the outcomes for the whole city (e.g., total number of expected buildings per damage grade), but this might not always be the case, particularly when looking at larger areas (e.g., other cities and towns around the Erft fault) where a greater variability of ground motion levels might be expected due, for example, to site or near-field effects. In such cases, considering all possible time periods of construction and numbers of storeys for each building (instead of sampling) would result in numbers of damaged buildings and losses that represent a mean value but not necessarily individually-realistic realisations, and for which the dispersion cannot be quantified. Moreover, it is interesting to note that, despite the stability of the aggregated results across realisations, the variability at the individual building level was indeed noticeable, an observation that is relevant when the spatial distribution of damage within the city is of interest. However, such results at the building level should be regarded with care, as they do not stem from a detailed study of individual buildings and building properties were still treated at the class level, firstly, by using a fragility model to describe the behaviour of the building and, secondly, by assigning a vulnerability class using the time period of construction as a proxy. As the fragility model was derived considering a group of buildings with similar characteristics and the assignment of vulnerability classes was done based on a sample of 631 buildings, the vulnerability models can only represent any particular building in a statistical sense.

The limitations of developing an exposure model based on VGI such as OpenStreetMap or the ODK dataset are clearly related to the reliability, accuracy and completeness of the data, as well as the difficulties associated with establishing links between spatially distributed objects with mismatches. Some of the potential limitations identified during this work were:

- The ODK dataset was retrieved from an open-contribution web service and, thus, the exact origin of the information regarding the year of construction within the dataset is unknown. Visual inspections using street-level and aerial imagery (e.g., Google Street View, Google Maps 3D view, Mapillary) suggest the existence of cases of both agreement and disagreement with respect to what can be observed and/or inferred from the imagery (e.g., adjustment of outliers in Table 5).
- Establishing an unequivocal relationship between polygons/points of one dataset and those of another is a challenging task that requires a series of decision-making criteria when the agreement is not perfect. The complete adequacy of the merging of the three datasets (OSM, ODK, NRW-WFS) cannot be guaranteed or even expected. The algorithm has room for improvement in dealing with complex cases, such as several ODK points being enclosed by one OSM polygon, or several NRW-WFS polygons intersecting one OSM polygon. A case-by-case human-based decision is not possible when dealing with large datasets.
- Cases have been observed of buildings that look like separate entities on street-level photos but are represented as one polygon in OSM and/or NRW-WFS. It appears that a polygon may sometimes represent one structure and, other times, one address. Cases have been found as well of (seemingly) vertically irregular structures (i.e., structures whose height is not the same for the whole of the building) often being represented as two separate polygons corresponding to the two sections of the building with different height. Any of these may lead to discrepancies with other sources (like censuses) on the overall number of buildings.
- The automatic processing of verbal descriptions of the functions and use of buildings for the purpose of assigning occupancy types was based on criteria developed to establish a relationship between keywords and the occupancy finally assigned. In many cases the description was not enough to fully and unequivocally interpret the kind of building under analysis. This means that the total number of buildings per occupancy type is likely to contain inaccuracies.

**Fig. 20:**
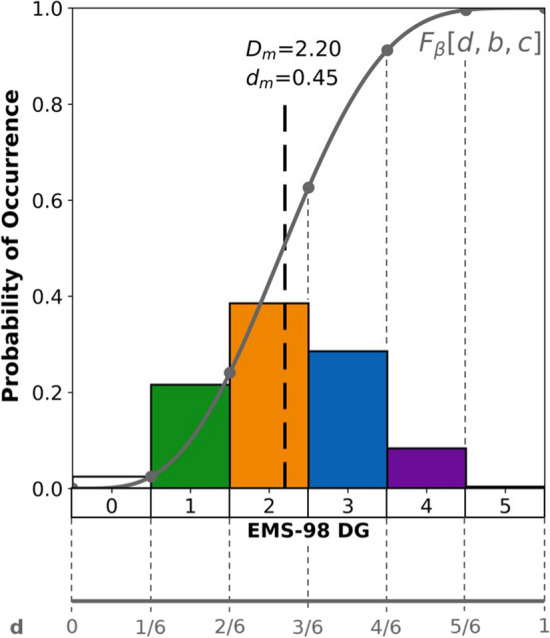
Example of the equivalence between the EMS-98 discrete damage grades, the normalised continuous [0, 1] domain of damage *d* and the mean damage grades in each of the two domains (*D*_m_ and *d*_m_, red). Colour bars show the probabilities of occurrence of the EMS-98 damage grades. CDF of the corresponding Beta distribution shown in grey

**Fig. 21:**
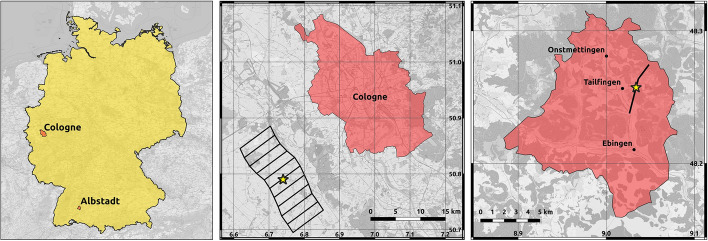
Location of Cologne and Albstadt within Germany (left) and location of the ruptures and epicentres (yellow stars) with respect to Cologne (centre) and the most damaged districts of Albstadt (right) (background map: OpenStreetMap)

The total number of residential buildings identified herein appeared as too large when compared against census data from previous years, the reason for which might lie in these potential inaccuracies and mismatches. The detailed evaluation of striking outliers (e.g., Table 5) and the consistency/plausibility checks carried out in this work (e.g., Figs. 10, 12, 20) were fundamental in this context to identify potential issues with the sources and the assumptions made.

Comparison against the consequences of past earthquakes is very useful in this sense as well, even when the ground motions, exposure and vulnerability are not exactly the same. Notwithstanding the differences and the associated uncertainties, damage results obtained herein for Cologne are reasonable when compared with damage statistics for the 1978 Albstadt earthquake, as was shown.

Our results for the earthquake scenario in Cologne show that around half of the 169,471 buildings identified as residential are expected to suffer from some degree of damage: 27% are expected to suffer from EMS-98 DG1, 12% from DG2, 5% from DG3, 2% from DG4, and 0.3% (around 400 buildings) from DG5. It is noted that these numbers result from the aggregation of probabilities for all residential buildings in the city, some of which may be quite small but whose sum builds up in virtue of the large numbers of buildings involved. Even if the most likely damage appears to be of a light-to-moderate nature, it can result in large economic costs and even injury, and should thus not be overlooked. For this scenario, more severe degrees of damage are expected mostly around the central area of Cologne, west of the river, and further west, while lighter damage is expected in the eastern area of the city. The possibility of studying the spatial distribution of damage at such high-resolution is a key advantage of working at the building-by-building level and is particularly relevant in so many cities around the world in which the downtown areas tend to be older (and more vulnerable) and the suburbs newer.

While the focus herein has been placed on a damage estimation due to the occurrence of a scenario earthquake, which allows for the quantification of potential losses due to one specific event and, consequently, the planning of post-earthquake emergency response, the use of a building-by-building exposure model is not limited to such a calculation and can be used for larger scale regional probabilistic risk analyses as well. Apart from the question on the availability of data for larger regions, the challenge in that case lies in the identification of a resolution with which to carry out the analyses that is appropriate for the available computational resources while providing sufficiently accurate results (e.g., Dabbeek et al. 2021), and/or the development of strategies to spatially segment the calculations in a useful and meaningful way. Some critical issues in this regard are the size of the target region and the complexity of the hazard model to be used, particularly in terms of number of end branches of the source/ground motion model logic tree. The quantification of the benefits and costs associated with the use of a building-by-building model with a Monte Carlo component for probabilistic risk assessments should thus be the focus of studies in the near future.

## Data Availability

Data sources used in this analysis are available from the references cited in the paper.

## Appendix A

The fragility model of Raschke (2003) was developed to be compatible with the EMS-98 macroseismic intensity scale in the sense that it yields the distribution of damage at each building site associated with each intensity *I*, as defined in the scale. At the same time, Raschke (2003) used empirical damage data from past earthquakes to evaluate the appropriateness of the adopted distributions and to derive a model of the uncertainty in the damage grade as a function of the mean damage grade *D*_m_. The latter is the average of all possible damage grades *i* weighted by their probability of occurrence, as shown in Eq. (2).

2 $$D_{m} = \mathop \sum \limits_{i = 0}^{5} P\left[ {DG_{i} } \right] \cdot i$$

The model comprises two main steps, the first one related to determining *D*_m_ and a second one in which *D*_m_ is converted into a set of probabilities of observing each of the possible damage grades from DG0 through DG5. A key aspect of the procedure is that it works simultaneously in two different domains: the 0 to 5 domain of the EMS-98 scale (to which *D*_m_ belongs) and a 0 to 1 domain that represents a normalised continuous version of the former, which is identified by the lower-case *d*. The mean damage grade is first determined in this normalised domain and assigned the name *d*_m_ as per Eq. (3):

3 $$d_{m} = \frac{{\tanh \left[ {0.0088 \cdot \left( {I_{k} - C} \right)^{2} + 0.2227 \cdot \left( {I_{k} - C} \right) - 2.4167} \right]}}{2} + 0.5$$

where *C* is the vulnerability index and *I*_k_ is the adjusted intensity, calculated as a function of the EMS-98 intensity, *I*, as

4 $$I_{k} = I + \frac{{\tanh \left( {3.3 \cdot I - 3.3 \cdot 6.5} \right)}}{2} + 0.5$$

The mean damage *d*_m_ in the [0,1] normalised range is transformed into the corresponding mean damage grade *D*_m_ in the 0 to 5 continuous domain of the EMS-98 damage scale as per Eq. (5):

5 $$D_{m} = \left\{ {\begin{array}{*{20}l} {0,} \hfill & {d_{m} < 1/12} \hfill \\ {6 \cdot \left( {d_{m} - 1/12} \right),} \hfill & {1/12 \le d_{m} \le 11/12} \hfill \\ {5,} \hfill & {d_{m} > 11/12} \hfill \\ \end{array} } \right.$$

In a second step, *D*_m_ is converted into probabilities of occurrence of individual EMS-98 damage grades DG_*i*_ by means of a Beta distribution, which has been deemed appropriate by Raschke (2003) and others (e.g., Lallemant and Kiremidjian 2015) to represent the distribution of earthquake damage. Thanks to the equivalence between *d* in the [0,1] continuous domain and the discrete EMS-98 damage grades DG_*i*_ established by Raschke (2003), the probability of observing each damage grade *i* is calculated as the probability of the damage grade *d* lying in between the lower (*d*_L_) and upper (*d*_U_) bounds defined in Table 11. This translates into a definite integral of the probability density function (PDF) of a Beta distribution with shape parameters *b* and *c*, f_ß_[*d*, *b*, *c*], which can be written in terms of the corresponding cumulative density function (CDF), F_ß_[*d*, *b*, *c*], as shown in Eq. (6):

6 $$P\left[ {\left. {DG_{i} } \right|I,C} \right] = \int_{{d_{L} \left( i \right)}}^{{d_{U} \left( i \right)}} {f_{\beta } \left[ {d,b,c} \right]dd = F_{\beta } \left[ {d_{U} \left( i \right),b,c} \right] - F_{\beta } \left[ {d_{L} \left( i \right),b,c} \right]}$$

**Table 11:** Equivalence between the damage grade *d* in the [0, 1] domain and the discrete EMS-98 damage grades DG_*i*_ according to Raschke (2003), to be used in Eq. (6)

EMS-98 damage grade DG_*i*_			*i* = 0	*i* = 1	*i* = 2	*i* = 3	*i* = 4	*i* = 5
Damage grade *d* in [0,1] domain	Upper bound	*d*_U_	1/6	2/6	3/6	4/6	5/6	1
	Lower bound	*d*_L_	0	1/6	2/6	3/6	4/6	5/6

The left-hand side of Eq. (6) explicitly states that the resulting probability of observing DG_*i*_ is a function of the intensity *I* and the vulnerability index *C*. These two variables are implicit in the rest of the equation through the fact that the two shape parameters *b* and *c* are calculated from the mean (*d*_m_) and standard deviation (*σ*_*d*_) of the damage grade *d* in the [0,1] domain. The latter is calculated as per Eq. (7):

7 $$\sigma_{d} = \sqrt {0.00212461 \cdot \sigma_{D}^{4} + 0.02296389 \cdot \sigma_{D}^{2} }$$

where *σ*_*D*_ is the standard deviation of the damage grade *D* in the continuous [0,5] domain which, based on a regression analysis carried out on observed data from past earthquake damage, Raschke (2003) suggests can be estimated by Eq. (8) as a function of *D*_m_:

8 $$\sigma_{D} = 0.4401 \cdot \left[ {D_{m} \cdot \left( {5 - D_{m} } \right)} \right]^{0.4358}$$

The shape parameters *b* and *c* of the Beta distribution are then calculated as per Eq. (9) and (10):

9 $$b = d_{m} \cdot \left[ {\frac{{d_{m} }}{{\sigma_{d}^{2} }} \cdot \left( {1 - d_{m} } \right) - 1} \right]$$

10 $$c = \left( {1 - d_{m} } \right) \cdot \left[ {\frac{{d_{m} }}{{\sigma_{d}^{2} }} \cdot \left( {1 - d_{m} } \right) - 1} \right]$$

Figure20 shows, as an example, the probabilities of the EMS-98 damage grades resulting from the model when *d*_m_ from Eq. (3) is 0.45 and depicts the equivalence between the two domains described above. Table 2 in the main text of the paper depicts the values of the vulnerability index *C* used in Eq. (3). The case in which *C* is agnostic to the number of storeys of Raschke (2003) is shown only for reference, as the values dependent on the number of storeys were actually adopted for the present work.

## Appendix B

Number of buildings within the administrative boundaries of the city of Cologne classified as per occupancy type resulting from the present study, following GEM’s Building Taxonomy v2.0 (Brzev et al. 2013), with certain adaptations (Table 12).

**Table 12:** Building stock of the city of Cologne per occupancy type as per the present study

Code	Description	Number
AGR	Agriculture, unknown type	157
AGR1	Agricultural storage	31
AGR2	Animal shelter (barn, stable, zoo building)	249
AGR3	Agricultural processing	1273
ASS	Assembly, unknown type	183
ASS1	Religious gathering (church, monastery)	968
ASS2	Arena	3
ASS3	Auditorium, cinema, concert hall	57
ASS5	Club house	245
ASS6	Cemetery	89
ASS7	Exhibition hall	40
COM	Commercial and public, unknown type	23,664
COM1	Retail trade	3018
COM2	Wholesale trade and storage (warehouse)	1479
COM3	Offices, professional/technical services	4648
COM5	Entertainment	82
COM6	Public building	138
COM7	Parking	1304
COM9	Railway station	35
COM11	Recreation and leisure	611
COM14	Hotel	616
COM15	Restaurant	344
COM16	Bank	79
COM17	Post	5
COM18	Gas station	198
EDU	Education and research, unknown type	252
EDU1	Pre-school	922
EDU2	School	820
EDU3	College and university, offices and/or classrooms	853
EDU4	Research facilities and/or labs	47
EME	Emergency, unknown type	146
EME1	Police	24
EME2	Firefighters	68
GOV	Government, unknown type	2
GOV1	Administration	129
GOV3	Town hall	9
GOV4	Diplomatic mission	4
GOV6	Court house	7
GOV7	Tax and customs	47
GOV8	Prison	55
IND	Industrial, unknown type	663
IND1	Heavy industrial (oil, petrochemical, timber, etc.)	22
IND2	Light industrial (factories, textiles, breweries, etc.)	1,662
IND3	Company building within industrial complex	50
IND6	Container	6
LIF	Buildings related to lifelines, unknown type	23
LIF1	Water	73
LIF2	Electricity	1434
LIF4	Sewage treatment	103
LIF5	Waste disposal	90
MED	Healthcare, unknown	247
MED1	Hospital	114
MED2	Sanatorium	17
MIX	Mixed occupancy: any mixed that is not MIX1	5565
MIX1	Mixed occupancy: residential and commercial	22,083
OTH	Other occupancy type	38
OTH1	Non-occupied	18
OTH2	Garage	63,372
RES	Residential, unknown type	11,674
RES1	Single dwelling	134,363
RES3	Temporary	1024
RES4	Institutional housing	327
TRA	Buildings related to traffic and transportation	2
TRA1	Maritime traffic	33
TRA2	Bus traffic	2
TRA3	Railway traffic	163
TRA4	Subway line	17
TRA5	Flight traffic	36
TRA6	Road	22
TRA7	Ropeway	3
UNK	Unknown	226

## Appendix C

The Albstadt earthquake of 1978 was reproduced for the purpose of enabling the comparison of its damage statistics against the damage estimated for the Cologne scenario studied herein. The two most affected districts of Albstadt for which damage data was available were considered: Tailfingen and Onstmettingen. Figure 21 shows the location of these districts with respect to the modelled rupture and epicentre.

The trace and characteristics of the Albstadt fault were retrieved from the European Database of Seismogenic Faults (Basili et al. 2013) and the OpenQuake engine (Pagani et al. 2014) was used to generate all possible ruptures on this fault with an aspect ratio of 1 that would cause an earthquake with *M*_w_ = 5.2 (GCMT), according to the Wells and Coppersmith (1994) magnitude scaling relations. Assuming the hypocentre to be located at the centre of the rupture, the rupture resulting in the hypocentre closest to that of Leydecker (2011) was selected (48.2567°N, 9.0267°E, 7 km depth), as it was the most consistent with the trace of the Albstadt fault in the European Database of Seismogenic Faults. According to the Global Centroid Moment Tensor (GCMT) project (Dziewonski et al. 1981; Ekström et al. 2012) and Turnovsky (1981), this was a strike-slip earthquake.

According to the German National Annex to Eurocode 8 (DIN EN 1998-1/NA:2011-01 2011), the area affected by the Albstadt earthquake is located on soil class R, that is, a site characterised predominantly by rocks. Detailed analysis by Schwarz et al. (2008) indicates that the district of Tailfingen lies mostly over soil class B-R, though classes C-R and C-S are also present. In these labels, R stands for predominantly rocks with expected thickness of sediments smaller than 25 m, while S corresponds to sediment basins with thicknesses of 100 m and larger. Soil class B is defined as loose soils with *v*_S25_ (the equivalent of *v*_S30_ but in the topmost 25 m instead of 30 m) in the range 350–800 m/s. Soil class C describes fine-grained soils with *v*_S25_ in the range 150–350 m/s. Based on these observations, ground motions in Tailfingen were calculated using *v*_S30_ = 250 m/s with Z_1_ = 100 m, representing the C-S sedimentary basin condition, and *v*_S30_ = 450 m/s with Z_1_ = 25 m, representing the B-R condition.

According to Porro and Schraft (1989), most of the buildings in the district of Onstmettingen lie either over alluvial deposits or slope debris, though some lie as well over chalk and marl rocks. As no further information was available, the same two alternative site conditions as for Tailfingen were considered for Onstmettingen as well.

## Appendix D

Figure 19 in the main text was generated through the following process:

1. Probabilities of occurrence of each damage grade were retrieved for all 2,000 realisations of the 39,183 buildings for which either only the number of storeys was known or nothing was known.
2. The standard deviations of the probabilities across all 2,000 realisations were calculated.
3. The sum of the standard deviations of the probabilities for damage grades 0 through 5 were added up (so as to have one value of dispersion per building).
4. 11,323 buildings with the summation of the standard deviations smaller than 1E-14 were discarded. Lack of variability in buildings for which Monte Carlo simulations were run can be due to the options of potential time periods of construction (and number of storeys, when relevant) to be assigned being too few. This happens when only a few buildings in the neighbourhood are run (i.e., few random buildings), the buildings with known properties are such that the possible left combinations are reduced, etc.
5. The remaining 27,860 buildings were ordered from smaller to higher sum of the standard deviations and plotted in that order from left to right (all six plots show the buildings in the same order).

## Code availability

The code will not be made available.
